# Synthesis of Amorphous Graphene and Graphene Oxide Analogues

**DOI:** 10.1021/jacs.5c00548

**Published:** 2025-03-25

**Authors:** Tomoki Sakuma, Ryoichi Sato, Akihiro Yamaguchi, Hiroaki Imai, Noriyoshi Arai, Yuya Oaki

**Affiliations:** † Department of Applied Chemistry, Faculty of Science and Technology, Keio University, 3-14-1 Hiyoshi, Kohoku-ku, Yokohama 223-8522, Japan; ‡ Department of Mechanical Engineering, Faculty of Science and Technology, Keio University, 3-14-1 Hiyoshi, Kohoku-ku, Yokohama 223-8522, Japan

## Abstract

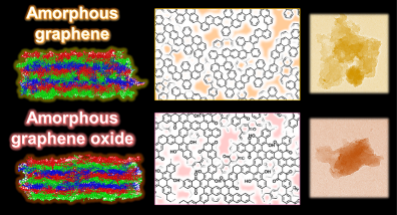

Graphene and graphene oxide (GO) are promising two-dimensional nanomaterials. An ultimate goal is to achieve large-scale bottom-up syntheses of perfect graphene and GO. However, controlled syntheses of perfect graphitic structures still remain challenges in chemistry and materials science. Moreover, amorphous types have not received much attention. The present work shows syntheses, structures, and applications of amorphous graphene and GO analogues alternative to the ideal ones. The simultaneous multiple reactions of two conjugated monomers provide amorphous conjugated polymer networks containing low-crystalline graphitic domains and their stacking. The stacked amorphous graphene and GO are exfoliated into thin nanosheets including few-layers and monolayers. Moreover, *in situ* syntheses of the amorphous GO analogues are applied to obtain a reinforced plastic with high mechanical strength. The present work implies that various functional nanocarbons can be designed and synthesized by tailored combinations of conjugated monomers.

## Introduction

**Figure 1:**
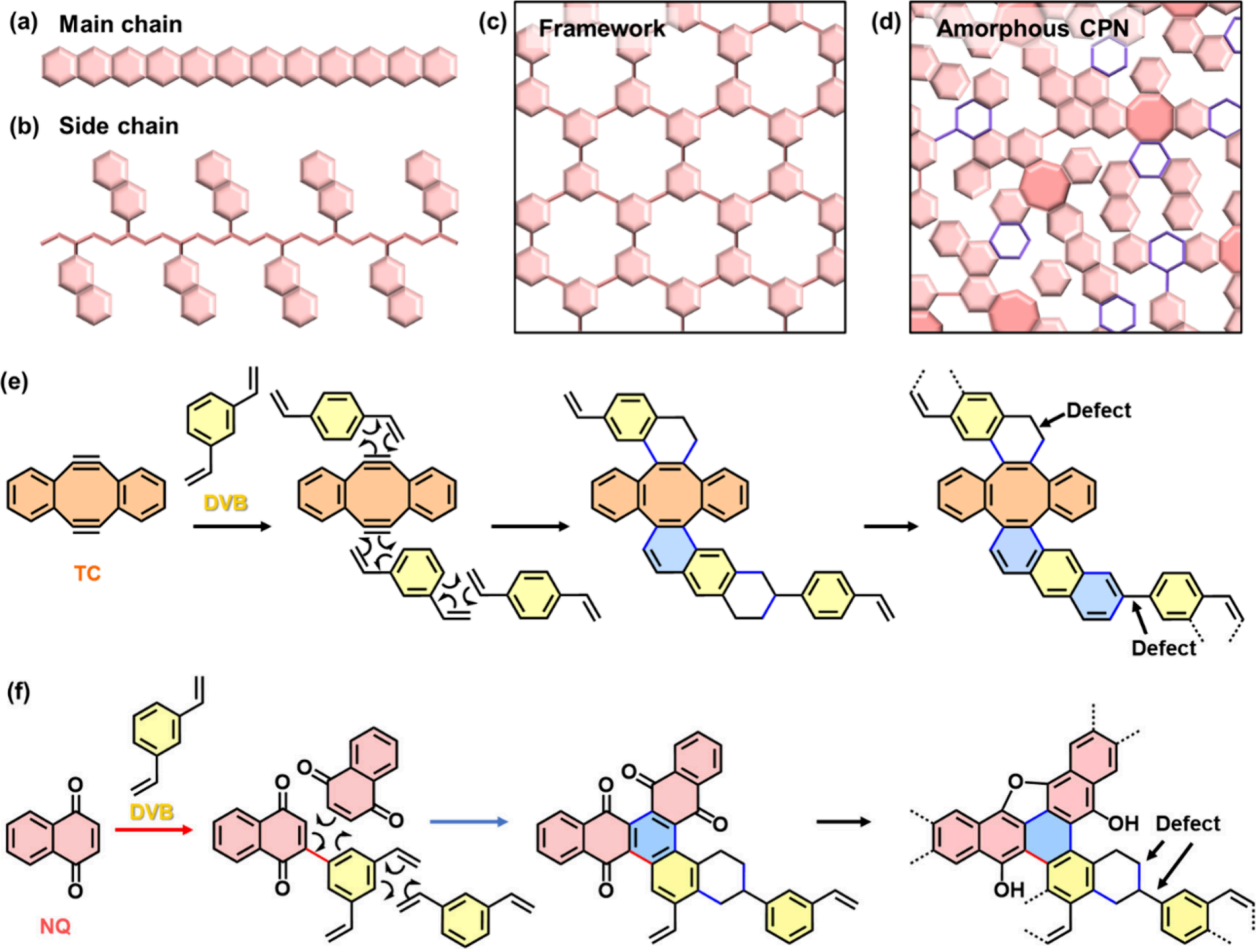
Design and synthesis of amorphous graphene and GO analogues based on CPNs. (a–d) Polymerized structures of π-conjugated monomers (pink hexagonal and octagonal plates): (a and b) Main- (a) and side- (b) chain types. (c) Framework types (MOF and COF). (d) Amorphous CPN containing nonconjugated moieties (purple sticks and colorless frames). (e and f) Synthetic schemes of TC-DVB (e) and NQ-DVB (f).

Two-dimensional (2D) materials, such as layered structures, few-layers, and monolayers, have attracted much interest.^1−11^ In general, ultrathin nanostructures are prepared by liquid-phase exfoliation of the precursor layered materials. Bottom-up synthesis is another route to obtain nanosheets. 2D graphitic nanocarbons are synthesized by various bottom-up approaches.^12−19^ In particular, syntheses of graphene analogues with the perfect honeycomb structure, such as graphene nanoribbons, have been extensively studied in the field of synthetic chemistry.^20−24^ On the other hand, the highly crystalline graphitic layer is not always needed for the applications, such as fillers for reinforced materials and porous materials for gas separation and adsorption. Here, we focused on synthesis and application of amorphous graphene and GO analogues, as a new family of nanocarbons. If π-conjugated moieties are extended with sufficient conjugated monomers in the amorphous conjugated polymer networks (CPNs) (Figure 1), the amorphous graphene and GO analogues can be designed and synthesized by the polymerization.

A variety of covalent and noncovalent approaches have been studied to organize functional π-conjugated molecules into higher ordered structures. Main- and side-chain types are the conventional polymerized structures (Figures 1a and 1b). In recent years, π-conjugated monomers have been organized in crystalline frameworks, such as metal- and covalent-organic frameworks (MOFs and COFs) (Figure 1c). In contrast to these ordered architectures, the low-crystalline disordered structures were not fully studied in previous works (Figure 1d). The reports on CPNs have gradually increased in the last two decades.^25−30^ Our group has focused on “amorphous” CPNs (Figure 1d).^31−36^ Simultaneous reactions of the multiple conjugated monomers form randomly networked low-crystalline polymers and their stackings. In amorphous CPNs, the nonconjugated moieties, such as single bonds and nonbenzene rings, are allowed to lower the crystallinity providing the structural flexibility (purple-framed colorless rings and octagonal rings in Figure 1d). In our previous reports, the amorphous CPNs containing the redox-active moieties, such as quinones, were applied to supercapacitors and electrocatalysts.^32−34^ The present work shows extension of the conjugation length and expansion of the graphitic domains to obtain amorphous graphene and GO analogues (Figures 1d–1f). The synthetic approach and targeted structure are different from those realizing perfect graphene in the earlier works.^12−24^

Structures and properties of amorphous 2D materials were theoretically studied to explore the potential properties originating from the low-crystalline nature.^37−41^ Amorphous monolayers of carbon and boron nitride were deposited on substrates by chemical vapor deposition.^42,43^ The laser irradiation to polyimide provided the amorphous graphene under inert atmosphere.^44^ The amorphous graphitic carbon, around 10 nm in thickness, was synthesized via the nanotubes.^45^ However, sufficient expansion of the graphitic domains was not observed in the Raman spectrum. In addition, large-scale bottom-up syntheses of the amorphous graphene and GO were not achieved in previous works. In the present work, amorphous CPNs containing sufficient graphitic domains were synthesized and exfoliated into the nanosheets. Moreover, a reinforced plastic was obtained by *in situ* syntheses of the amorphous GO in the presence of matrix polymer. Based on the present work, a variety of amorphous graphene and GO analogues containing heteroatoms can be designed and synthesized by combinations of monomers. Three-dimensional (3D) nanocarbon materials with the specific porosity and metal loading have been extensively studied in recent years.^46−48^ Our design strategy can be applied to the structural control of not only 2D but also 3D nanocarbons for their further functionalization.

## Results and Discussion

### Molecular Design of Amorphous CPNs as Graphene and GO Analogues

The amorphous CPNs with the low-crystalline graphitic domains were designed by reaction of 1,3- or 1,4-divinylbenzene (DVB, mixture of *meta* and *para*) with the dienophiles, such as 5,6,11,12-tetradehydrodibenzo[*a*,*e*]cyclooctene (TC) for graphene analogue and 1,4-naphthoquinone (NQ) for GO analogue (Figures 1e and 1f). Diels–Alder (DA) reaction of TC and DVB expands the π-conjugated plane. If the DA reaction proceeds between the DVB moiety and free DVB, the nonconjugated moieties, such as the C–C single bond and cycloalkane ring, are partially introduced as the defect in the network (colorless rings in Figure 1e). The nonconjugated moieties, represented by the blue stick and rings in Figure 1d, provide the structural flexibility in the CPN. On the other hand, the conjugation can be recovered by the spontaneous 1,3-hydrogen transfer step after the DA reaction.^49^ The consecutive DA reactions of NQ and DVB molecules generate the π-conjugated plane (Figure 1f). The oxidized states are introduced in the amorphous CPN by C=O groups of NQ. In this manner, amorphous graphene and GO analogues are designed by the consecutive DA reactions providing the π-conjugated plane and nonconjugated moieties in the CPNs.

Prior to selection of TC and NQ, the other dienophiles, such as naphthalene (NP), anthracene (AT), tetracene (naphtacene, NC), and anthraquinone (AQ), were used to compare the reactivity. A high-throughput screening was carried out to select the combinations of the reactive monomers (Figure S1). The reaction with DVB was observed only for TC and NQ in the screening experiment.

### Synthesis of TC-DVB and NQ-DVB

**Figure 2:**
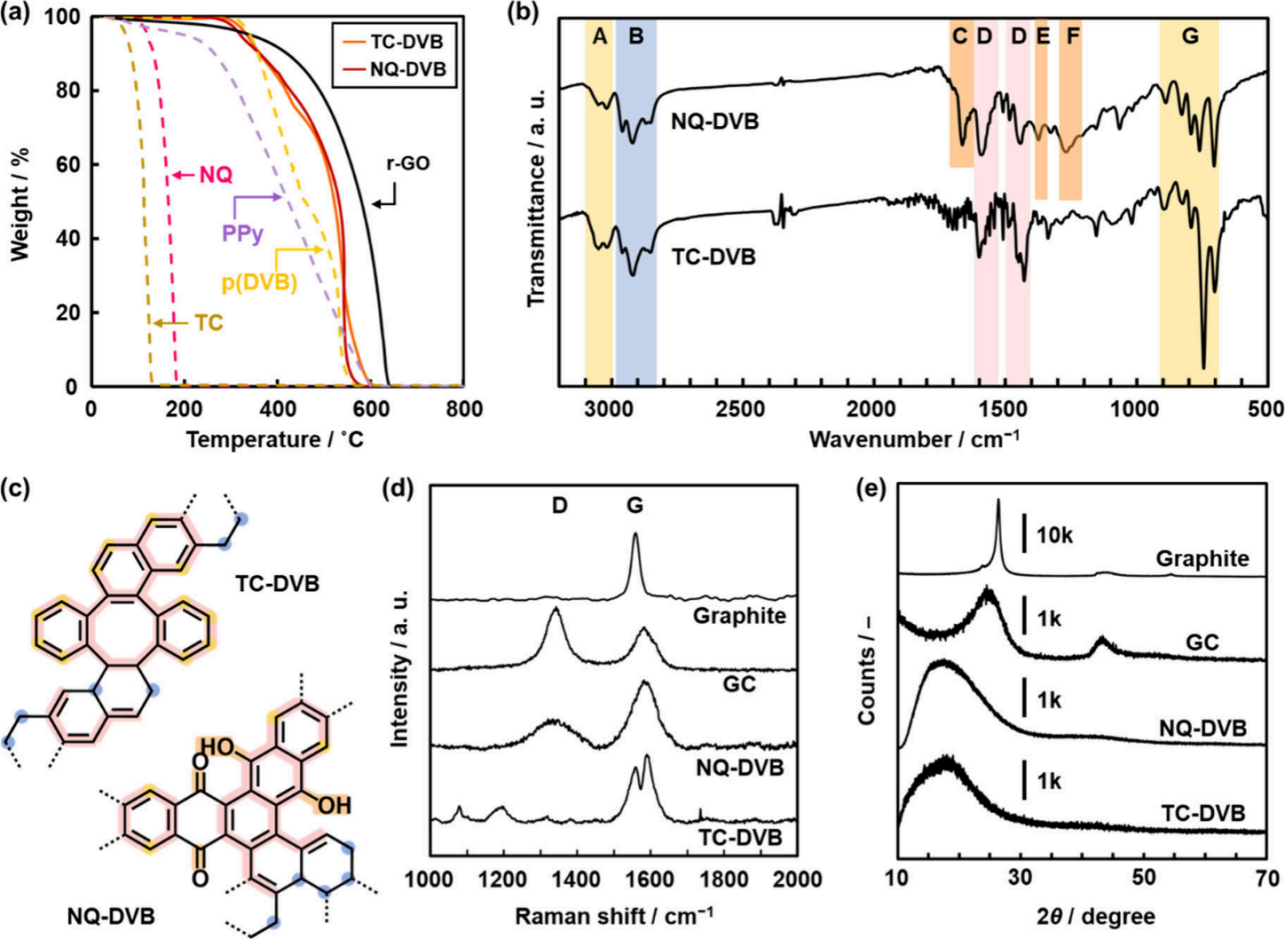
Structural analyses of TC-DVB, NQ-DVB, and their reference samples. (a) TG curves (heating rate: 10 K min^–1^). (b) FT-IR spectra. (c) Partial molecular structures containing the characteristic bonds observed in the FT-IR spectra. (d) Raman spectra. (e) XRD patterns.

The equimolar mixture of TC and DVB or NQ and DVB was heated at 200 °C for 1 h in toluene under microwave irradiation. The resultant solid was vacuum-dried at 200 °C for 16 h to remove the remaining monomers and oligomers. The detailed procedure was described in the Supporting Information. The black precipitates were obtained with a yield of 72% for TC-DVB and 70% for NQ-DVB. TC-DVB and NQ-DVB showed the weight loss around 500 °C on the thermogravimetry (TG) curves under air atmosphere (Figure 2a). The weight-loss temperature was higher than those of the solid TC and NQ monomers. The weight loss with combustion was observed in the temperature range higher than that of the reference linear polymers, such as polypyrrole (PPy) and polymerized DVB (pDVB). The weight-loss temperature is comparable to that of a commercial reduced GO (r-GO) as a graphene. TG analysis of PPy and rGO under inert argon (Ar) atmosphere showed the thermal decomposition around 200 and 400 °C, respectively (Figure S2). The thermal stability of the network rGO is higher than that of chain PPy under an Ar atmosphere. Whereas the thermal decomposition of TC-DVB and NQ-DVB was observed around 400 °C similar to that of rGO, the weight loss was larger than that of rGO. The defect domains based on sp^3^ carbon are preferentially decomposed with heating. These TG analyses imply the formation of polymerized network structures.

The following broadened absorption was observed in the Fourier-transform infrared (FT-IR) spectra of TC-DVB and NQ-DVB (Figures 2b and 2c): C–H stretching vibrations of an aromatic ring around 3050 cm^–1^ (band A) and methylene around 2950 cm^–1^ (band B), C=O stretching vibration of a benzoquinone moiety around 1670 cm^–1^ (band C) for NQ-DVB, C=C stretching vibrations of an aromatic ring in the range of 1400–1600 cm^–1^ (band D), O–H and C–O stretching vibrations of a hydroquinone moiety around 1370 and 1260 cm^–1^ (bands E and F) for NQ-DVB, respectively, and C–H in-plane bending vibration of an aromatic ring (band G). All of these chemical bonds are contained in the estimated partial structures (Figure 2c). The FT-IR spectrum was different from that of the monomers and pDVB (Figure S2). The peak broadening implies the polymerization. Formation of the graphitic network structures was supported by solid-state ^13^C nuclear magnetic resonance (NMR) spectroscopy (Figure S3).

UV–vis–NIR spectra show that the absorption edge was extended to around 1000 nm for TC-DVB and 1600 nm for NQ-DVB (Figure S4). In contrast, the monomers had absorption in the UV–vis region. The absorption in the NIR region indicates an extension of the conjugation length. Raman spectra of NQ-DVB showed the broadened D and G bands characteristic of the graphitic carbons at 1330 and 1590 cm^–1^, respectively (Figure 2d). As a reference, only the G band was observed for a commercial graphite. A commercial glassy carbon (GC) showed both D and G bands with an intensity ratio (*I*_G_/*I*_D_) of 0.69. The intensity ratio was *I*_G_/*I*_D_ = 2.37 for NQ-DVB. The Raman spectra indicate the extension of the π-conjugated network. The split peaks were observed for TC-DVB near the regions of the D and G bands (Figure 2d). Whereas low molecular-weight aromatic compounds show many sharpened peaks in the range of 1000–2000 cm^–1^,^50,51^ a number of the broadened peaks are observed for fullerenes and polycyclic aromatic hydrocarbons (PAHs) near the region of the D and G bands.^52−54^ These facts imply that the TC-DVB network consisted of PAH domains. As UV–vis–NIR spectra of TC-DVB showed the absorption edge shorter than that of NQ-DVB (Figure S4), the conjugation length of TC-DVB is shorter than that of NQ-DVB.

**Scheme 1:**
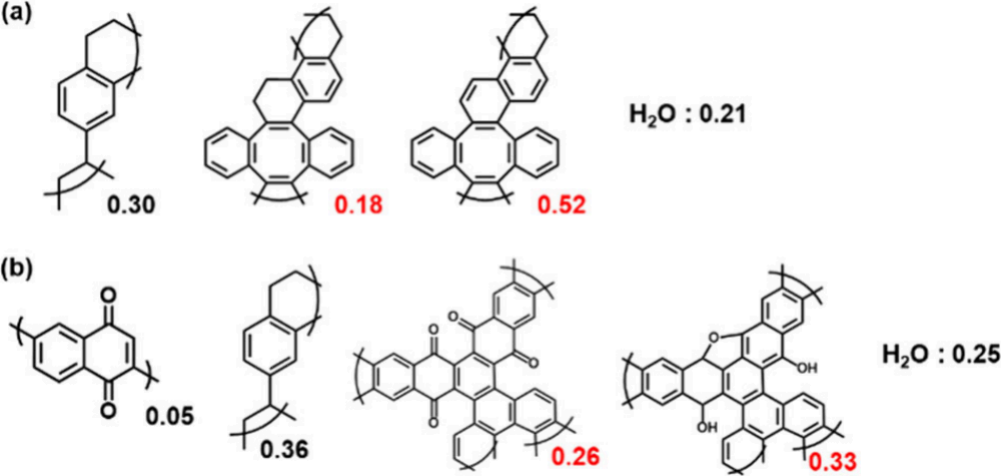
Estimated Unit Structures and Compositions of TC-DVB (a) and NQ-DVB (b) The red-color indexes correspond to the conjugated domains in the networks.

Based on these structural analyses, the composition and repeating unit of NQ-DVB and TC-DVB network structures were estimated using CHN elemental analysis (Scheme 1 and Table S1). The measured weight ratios of C, H, N, and the others were 86.5, 6.18, 0.43, and 6.89 for NQ-DVB and 92.7, 6.09, 0.12, and 1.10 for TC-DVB, respectively. The estimated structures were drawn based on the measured wight ratio of C, H, and N on the assumption that the others correspond to O (Scheme 1). The calculated weight ratio from the estimated structures was consistent with that of the measured ones within 0.5% (Table S1). Based on the composition, TC-DVB and NQ-DVB contained 59% and 70% of conjugated domains in the network (the red indexes in Scheme 1), respectively. On the other hand, the ratio was less than 34% for the other amorphous CPNs in our previous works.^33−35^ The conjugated domains can be expanded by the molecular design of CPNs.

### Stacking of TC-DVB and NQ-DVB with Amorphous Graphitic Domains

The stacking states of the amorphous CPNs were compared to the other graphitic carbons. X-ray diffraction (XRD) showed the broadened peak centered around 2θ = 18.0 ° for TC-DVB and 19.6° for NQ-DVB (Figure 2e). The peak originates from stacking of the amorphous graphitic domains. The corresponding lattice spacing, i.e., interlayer distance (*d*_0_), was calculated to be *d*_0_ = 0.49 nm for TC-DVB and 0.45 nm for NQ-DVB. A commercial graphite and r-GO showed sharpened peaks corresponding to *d*_0_ = 0.34 nm (Figure 2e and Figure S5). On the other hand, GC had the broadened peaks corresponding to *d*_0_ = 0.36 nm (Figure 2e). The larger *d*_0_ = 0.82 nm was observed for GO with the high-oxidized state (Figure S5). Based on these results, the TC-DVB and NQ-DVB networks are stacked with the spacing larger than that of the ideal graphitic structures. As these amorphous CPNs contained the nonplanar units, such as sp^3^ carbons, ether, and carbonyl groups (Scheme 1), the planarity is lower than that of the graphitic domains in the other nanocarbons. The conductivity of TC-DVB was measured using a pelletized sample of the powder with attaching metallic electrodes. However, the value of resistance was not obtained because of the high resistance. The π-conjugation and π–π stacking for sufficient conductivity are not fully achieved for the amorphous CPNs.

**Figure 3:**
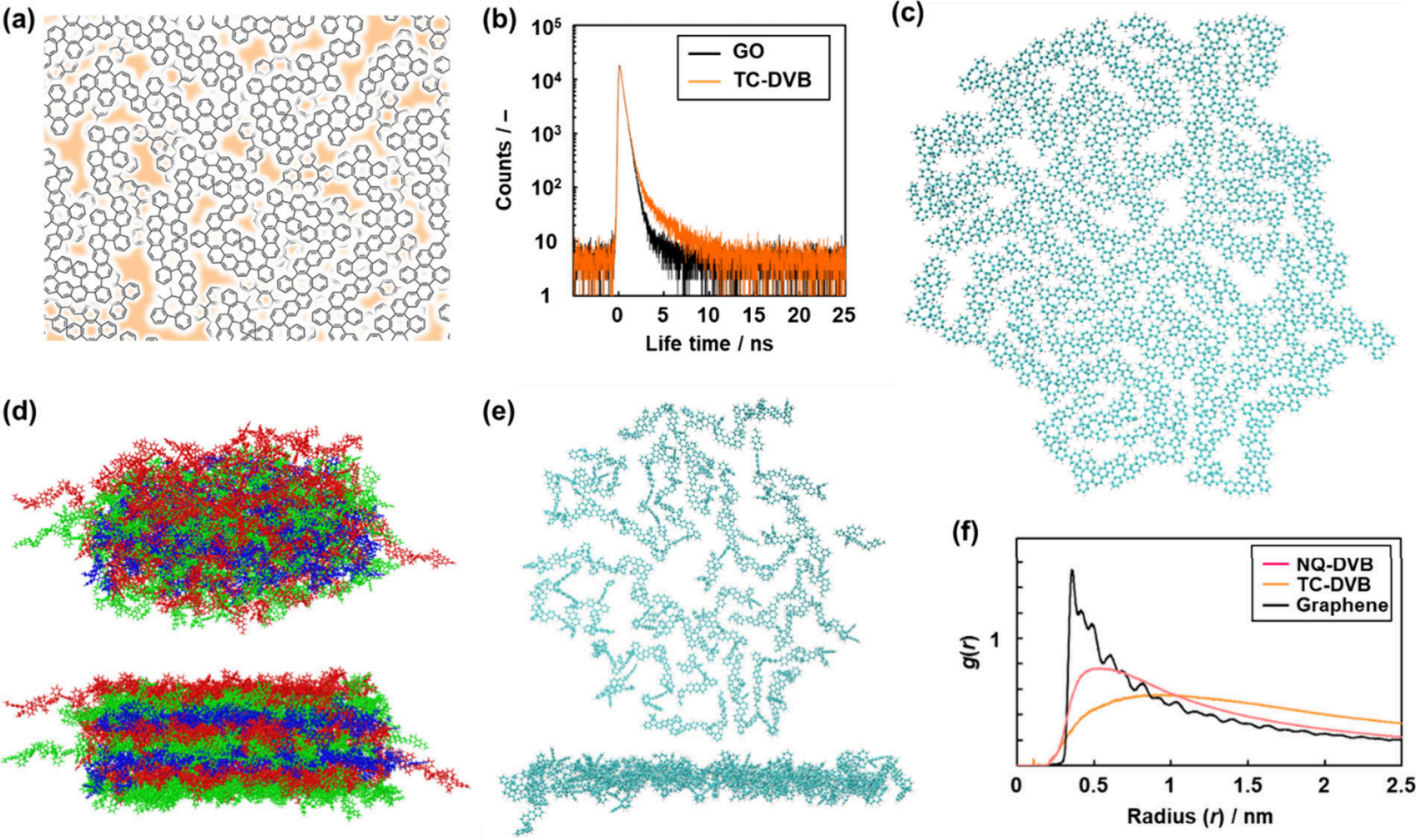
Structural analyses and MD simulation of the TC-DVB network and its stacking. (a) Schematic illustration of the network structures with the colored interspace corresponding to the free volume space. (b) PALS lifetime curves of TC-DVB and reference GO (see Table 1). (c) Original unit layer for the calculation. (d) Diagonal (upper) and side (lower) views of the stacked structure including second-to-eighth layers after the calculation. (e) Top (upper) and side (lower) views of the monolayer visualized in the stacking. (f) RDF curves of TC-DVB, NQ-DVB, and reference graphene. The same data for NQ-DVB are in Figure S8.

Based on these analyses, the network structures as the monolayer were illustrated for TC-DVB (Figure 3a) and NQ-DVB (Figure S8). These amorphous CPNs have specific voids in the network, as drawn by the colors in Figure 3a for TC-DVB (Figure S8 for NQ-DVB). In contrast, no such void is included in ideal graphene layer. The pore size and specific surface area originating from such void were not measured using the nitrogen gas adsorption technique (Figure S6). As the small pore is closed by the stacking of the network structures, gas molecules are not diffused and adsorbed in the pore. Instead of gas adsorption, positron annihilation lifetime spectroscopy (PALS) shows the specific lifetimes originating from the characteristic structures of nanocarbons.^55−60^ The structures of TC-DVB and NQ-DVB were compared with those of commercial graphite, r-GO, GO, and GC using PALS (Figure 3b, Table 1, and Figure S7). The lifetime curve was analyzed and approximated by the three components, τ_1_, τ_2_, and τ_3_ (Table 1). Based on the previous reports,^55−60^ these lifetimes (τ_1–3_) and their relative intensities (*I*_1–3_) are assigned to the following annihilation mechanisms of positronium: π electrons in the whole volume of the sample (τ_1_ ≈ 0.2 ns, *I*_1_), molecular-level surface of the graphitic carbon material depending on the functional groups and adsorbed species (τ_2_ ≈ 0.4 ns, *I*_2_), and free volume typically observed in polymer materials (τ_3_ ≈ 2 ns, *I*_3_).^55−60^ The relative intensity corresponds to the abundance ratio. Table 1 indicates that the *I*_1_ values of TC-DVB and NQ-DVB were comparable to those of r-GO and GO. The fact implies that the amorphous graphitic structure with sufficient π electrons is contained in the networks of TC-DVB and NQ-DVB. Whereas *I*_2_ of TC-DVB and NQ-DVB was smaller than that of r-GO and GO, the *I*_3_ values showed the larger values. The increase in *I*_3_ and decrease in *I*_2_ indicate the formation of the free volume space in TC-DVB and NQ-DVB. In TC-DVB and NQ-DVB, the free volume space corresponds to the colored area in Figure 3a (Figure S8). The PALS analyses support the formation of the proposed amorphous network structures with the free volume space.

**Table 1:** Summary of Three-Component Analyses for PALS

samples	τ_1_ (ns)	*I*_1_ (%)	τ_2_ (ns)	*I*_2_ (%)	τ_3_ (ns)	*I*_3_ (%)
graphite	0.126	12.8	0.354	85.7	1.634	1.5
r-GO	0.139	8.5	0.374	90.3	1.680	1.2
GO	0.131	8.4	0.384	90.5	1.740	1.1
GC	0.125	1.5	0.417	97.4	1.358	1.1
TC-DVB	0.136	7.4	0.383	87.9	2.260	4.7
NQ-DVB	0.094	6.3	0.357	84.6	2.178	9.1

The expanded networks and their stacked structures were constructed using molecular dynamics (MD) simulations (Figures 3c–3f and Figure S8). The unit layers of TC-DVB and NQ-DVB were prepared using a total of 5571 and 3958 atoms based on the compositions in Scheme 1, respectively (Figure 3c and Figure S8). As a reference, ideal graphene was prepared using 1015 atoms (Figure S9).

Ten layers were stacked with rotation of each layer in the in-plane direction to prepare the model. Cyclohexane molecules were filled in the edge space of the cell to avoid unrealistic deformation of the expanded layers. After the calculation, the second to eighth layers in the total 10 layers were visualized and color-coded by red, green, and blue (Figure 3d). In the diagonal view, the layers were roughly stacked with each other (upper panel in Figure 3d). The stacked structure was observed in the side view (the bottom panel in Figure 3d). When one TC-DVB layer was visualized in the stacked state (Figure 3e), the layer was not flat, but uneven. The similar uneven monolayer and its rough stacking were constructed for NQ-DVB (Figure S8). On the other hand, the reference graphene showed a flat structure and its stacking (Figure S9). The average thickness of the monolayers was calculated to be 0.94 ± 0.19 nm for TC-DVB and 0.53 ± 0.04 nm for NQ-DVB in the second to eighth layers (the lower panel in Figure 3e and Figures S8 and S10). The stacking periodicity of the layers, corresponding to the interlayer distance, was estimated from the radial distribution function (RDF) in the layers (Figure 3f). TC-DVB and NQ-DVB exhibited broadened RDF curves, whereas the RDF of graphene showed a sharpened peak. The peaks were observed in the horizontal *r* axis in the order of graphene, NQ-DVB, and TC-DVB. This trend in the RDF curves is consistent with the peak positions of the XRD patterns (Figure 2e). Based on the models, the interlayer distance was calculated by dividing the total thickness of the 10-layered structure by the number of layers. The calculated interlayer distances were 0.585 nm for TC-DVB, 0.458 nm for NQ-DVB, and 0.333 nm for graphene. The simulated and measured values are consistent with each other. In this manner, MD simulations supported the formation of layered structures based on the TC-DVB and NQ-DVB amorphous CPNs.

### Exfoliation of TC-DVB and NQ-DVB into the Nanosheets

**Figure 4:**
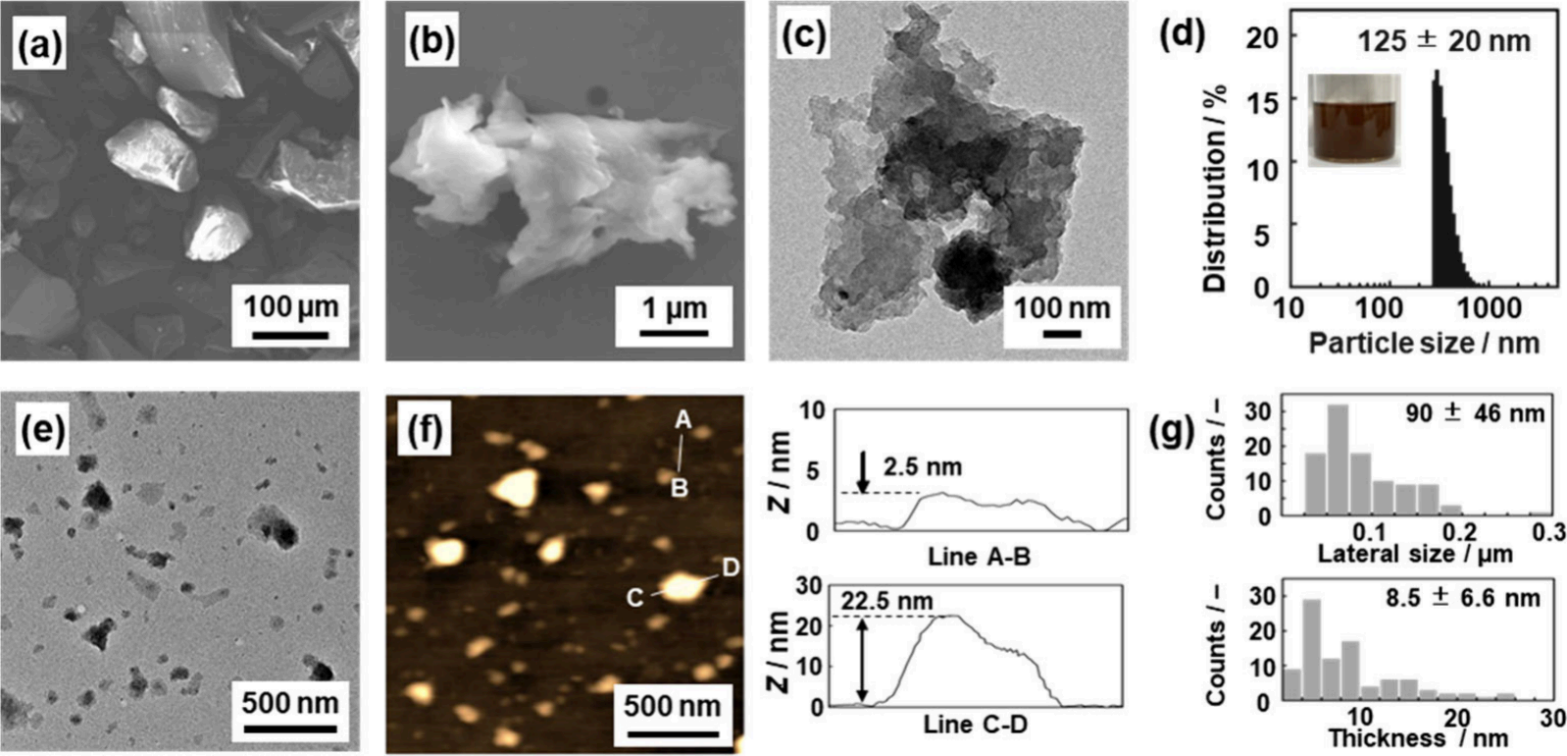
Morphology of TC-DVB before and after the exfoliation for 5 min (b and c) and 60 min (d–f). (a) SEM image after the synthesis. (b and c) SEM and TEM images after the exfoliation in chlorobenzene for 5 min, respectively. (d) DLS particle-size distribution and photograph of the dispersion liquid after the exfoliation for 1 h (inset). (e) TEM image. (f) AFM image (left) and representative height profiles (right) of the thinner (upper) and thicker (lower) nanoflakes. (g) Distribution of the lateral size (upper) and thickness (lower) estimated from TEM and AFM images, respectively. The same data for NQ-DVB are in Figure S11. The mean and standard deviation were calculated by all the observed particles including the smaller and larger ones.

The stacked structures of TC-DVB and NQ-DVB were exfoliated into thin nanostructures (Figure 4 and Figure S11). The bulk particles of around 100 μm in size were obtained after the synthesis (Figure 4a). The colloidal liquid with a brown color was formed with sonication of the bulk particles in chlorobenzene (inset of Figure 4d). After dispersion for 5 min, the sheet-like objects were observed on the images of scanning electron microscopy (SEM) and transmission electron microscopy (TEM) (Figures 4b and 4c). The exfoliation was carried out for 1 h. The average particle size in the dispersion liquid was 125 ± 20 nm for TC-DVB and 59 ± 15 nm for NQ-DVB as determined by dynamic light scattering (DLS) (Figure 4d). The average lateral size was 90 ± 46 nm for TC-DVB and 99 ± 52 nm for NQ-DVB based on the TEM images (Figure 4e). The sizes estimated from the TEM and DLS analyses are consistent with each other. No lattice fringes and ordered structures were observed on the high-resolution TEM images of the exfoliated NQ-DVB and TC-DVB nanosheets (Figure S12). The fact supports the formation of the amorphous interior as shown in Figure 3a. The nanoflakes are homogeneously dispersed without aggregation in the liquid phase. The average heights were 8.5 ± 6.6 nm for TC-DVB and 7.1 ± 6.0 nm for NQ-DVB based on the atomic force microscopy (AFM) images (Figure 4f).

**Figure 5:**
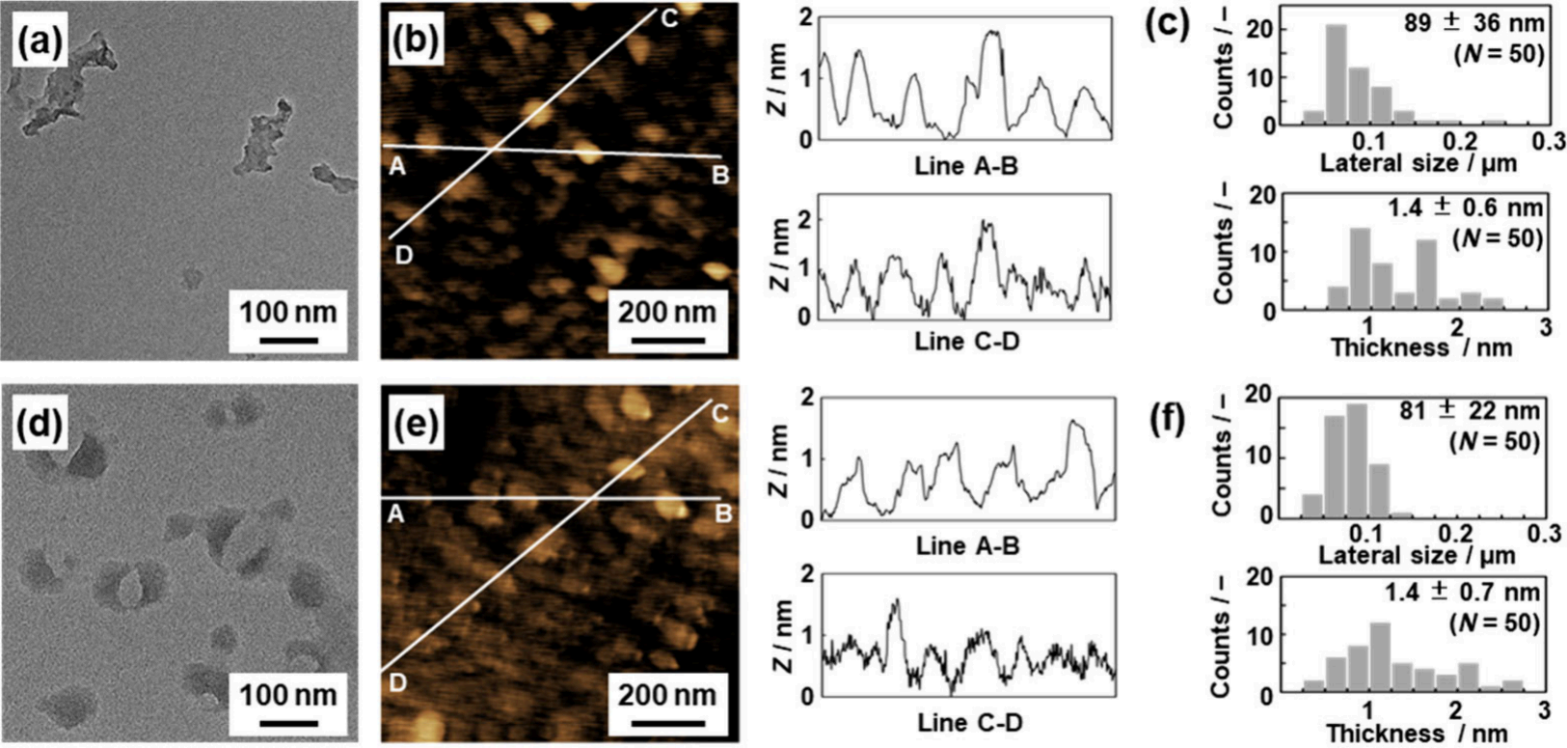
Thiner nanoflakes of TC-DVB (a) and NQ-DVB (d–f). (a and d) TEM images. (b and e) AFM images and height profiles. (c and f) Distribution of the lateral size (upper) and thickness (lower) estimated from TEM and AFM images, respectively.

When the colloidal liquids were centrifuged at 6000 rpm for 30 min, the thinner flakes around 1 nm were obtained with the removal of the thicker ones (Figure 5 and Figure S13). Based on the thickness estimated from the MD simulation (Figure S10), the monolayers and few-layers are selectively collected by purification using centrifugation. No structural changes in the molecular level were observed for the thinner flakes in the FT-IR and Raman spectra (Figure S13). On the other hand, the absorption edge was shifted to the shorter wavelength region (Figure S13). As the thin nanoflakes consisting of the flexible network structure are formed in the dispersion media, the blueshift is induced by shortening the effective conjugation length with the molecular motion.

These results indicate that the amorphous graphene and GO analogues were obtained by the exfoliation of the stacked structure with dispersion in chlorobenzene with sonication for 1 h. The low-crystalline nature of the amorphous CPNs contributes to enabling efficient exfoliation. The particle size control of the amorphous CPN was studied by the application of the precipitation polymerization method.^35^ The size control of the exfoliated nanosheets was achieved using the prediction models.^61^ The sizes of the stacked and exfoliated states can be controlled by these methods.

### Reinforced Plastic Using the Amorphous GO Analogue

**Figure 6:**
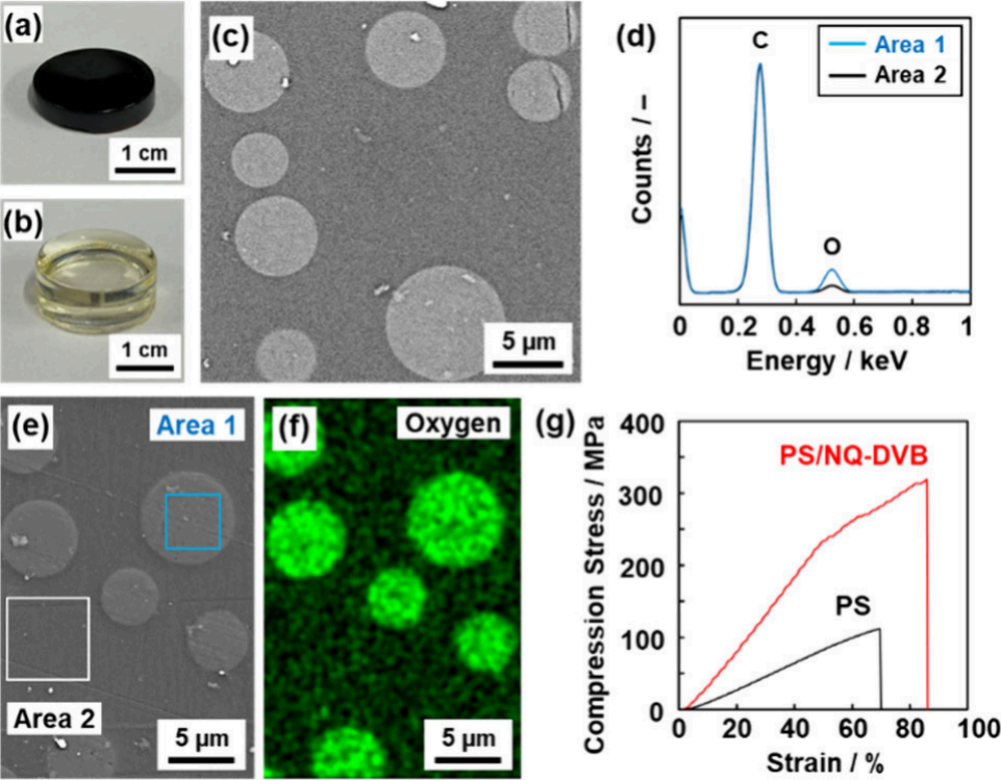
NQ-DVB-reinforced PS (PS/NQ-DVB (30%)). (a and b) Photographs of PS/NQ-DVB (a) and PS (b). (c) Cross-sectional BSE image of PS/NQ-DVB. (d) EDX spectrum of areas 1 (island, blue line) and 2 (sea, black line) on the cross section (normalized by the intensity of C). (e and f) Cross-sectional SE image (e) and elemental mapping of O (f). (g) Stress–strain curves of PS/NQ-DVB and PS for the compression experiment.

As a model case, NQ-DVB as the GO analogue was applied to reinforcing a plastic (Figure 6). Nanocarbons with graphitic structures, such as carbon fiber and r-GO, are used as fillers to prepare reinforced plastics.^62−64^ However, the concentration of the carbon fillers has the upper limitations, typically several percents, to preserve the homogeneous dispersion in the matrix polymers.^65^ The specific methods have been studied to achieve the high filler concentration.^66^ Here, the *in situ* synthesis of NQ-DVB as a filler was performed in the precursor solution containing polystyrene (PS) as a matrix polymer. NQ and DVB were dissolved in toluene containing PS (*M*_w_ = 1.92 × 10^5^). The toluene solution was poured in a mold and then heated at 200 °C for 5 h without sealing to evaporate the solvent. The initial weight ratio of NQ-DVB was set at 30 wt % to PS. The black colored PS resin was obtained after the evaporation of toluene (Figure 6a), whereas the transparent bulk one formed without addition of the monomers (Figure 6b). The fractured surface exhibited a dense body with the sea–island structure on the secondary electron (SE) and backscattered electron (BSE) images using SEM (Figures 6c and 6e). The bright island domain (area 1) showed a strong peak of oxygen (O) on the energy-dispersive X-ray (EDX) spectrum, whereas the peak was weak for the dark sea domain (area 2) (Figures 6d and 6e). In the EDX mapping, the peak of the O atom was detected on the sea domain (Figure 6f). Therefore, the bright and dark domains correspond to the NQ-DVB and PS, respectively. NQ-DVB in the composite had a similar structure as that prepared in the absence of PS (Figure S14). The actual concentration of NQ-DVB in the composite was 25.8 ± 8.8% based on the elemental analysis.

The resultant PS/NQ-DVB composite exhibited enhanced mechanical properties, namely, the compression strength (σ) maximum 342 MPa (average 318 ± 22 MPa, the sample number *N* = 8) and modulus 550 MPa (average 308 ± 122 MPa, *N* = 8) (Figure 6g and Figure S15). On the other hand, the compression strength and modulus of the PS prepared without NQ-DVB were σ_0_ = 116 ± 8 and 148 ± 28 MPa, respectively (Figure 6g and Figure S15). The compression strength of PS was enhanced by (σ/σ_0_ = ) 2.76 times with the infiltration of NQ-DVB. The reference composites were prepared from the PS solution with the dispersion of 1 wt % commercial GC, multiwalled carbon nanotube (CNT), and r-GO. These carbon fillers were not homogeneously dispersed even at 1 wt % in the toluene solution containing PS (Figure S16). The carbon fillers were aggregated in submillimeter scale, whereas the homogeneous sea–island structure was formed in micrometer scale for the PS/NQ-DVB composite. These reference composites were fragile and immediately fractured with applying the compression stresses around 5 MPa (Figure S16). The mechanical properties were weakened compared with those of pristine PS. Our PS/NQ-DVB showed both the highest reinforcement (σ/σ_0_) and compression strength (σ) compared with those of the other plastics reinforced by fillers in recent works (Table S2 and Figure S17).^67−77^

The homogeneous dense composites were not obtained at the initial NQ-DVB concentrations 10 and 20 wt % because the void was formed inside with the evaporation of toluene. Although the homogeneous and dense composites were obtained at the initial NQ-DVB concentrations 40 and 50 wt % (Figure S15), the mechanical properties lowered compared with those at 30 wt %. In this manner, PS was reinforced by the *in situ* synthesis of the initial NQ-DVB filler. Nanocarbons are generally used to enhance the mechanical properties with mixing and dispersing in matrix polymers.^62−66^ However, these carbon fillers are not easily dispersed at high concentrations in the matrix. For example, the mechanical strength was not improved with increasing the carbon concentration higher than 5–10 wt %.^78−84^ In the present work, NQ-DVB as an amorphous GO is directly synthesized in a solution containing PS as a matrix polymer. The *in situ* synthesis enables the high concentration of the filler comparable to that in polymer alloys. Polymer alloys based on the graphitic carbons are not obtained in previous works because the graphitic carbons are not directly synthesized in the presence of matrix polymers with the homogeneous dispersion. The present results imply that new lightweight reinforced plastics can be prepared by the *in situ* synthesis of the amorphous CPNs with the conjugated moieties in the matrix polymers.

## Conclusions

Amorphous graphene and GO analogues were designed and synthesized by extension of the π-conjugated framework and expansion of the low-crystalline graphitic domains in CPNs. The simultaneous multiple reactions of π-conjugated monomers provided amorphous CPNs through their random copolymerization. TC-DVB and NQ-DVB as the amorphous graphene and GO analogues were synthesized from the monomers with sufficient conjugated moieties. The stacked structures were exfoliated into the nanosheets, including monolayers and few-layers. A new reinforced plastic was prepared by *in situ* synthesis of NQ-DVB in the toluene solution containing PS. The sea–island structure of PS and NQ-DVB had the enhanced mechanical strength to the compression stress. Whereas design and synthesis of perfect graphene analogues were extensively studied, the amorphous types have not attracted much interest. The present work implies that new functional 2D nanocarbons can be synthesized based on the amorphous CPNs using diverse combinations of conjugated monomers.

## Supporting Information Available

Additional experimental methods, FT-IR, ^13^C NMR, Raman, UV–vis–NIR spectra, XRD patterns, elemental analysis, pore-size analysis, AFM images, TEM images, MD simulations, and mechanical properties of NQ-DVB, TC-DVB, and their reference materials (PDF)
