# Supplementary material for: Synthesis of Amorphous Graphene and Graphene Oxide Analogues
Source: J Am Chem Soc. 2025 Mar 25;147(13):11564–73. doi: 10.1021/jacs.5c00548 (PMC11969549; doi:10.1021/jacs.5c00548)
Supplement: Supplementary file 1 — ja5c00548_si_001.pdf [file ja5c00548_si_001.pdf]

Supporting Information

Synthesis of Amorphous Graphene and Graphene Oxide Analogues

Tomoki Sakuma,† Ryoichi Sato,‡ Akihiro Yamaguchi,‡ Hiroaki Imai,† Noriyoshi Arai,‡ Yuya Oaki*,†

† Department of Applied Chemistry, Faculty of Science and Technology, Keio University, 3-14-1 Hiyoshi, Kohoku-ku, Yokohama 223-8522, Japan.

‡ Department of Mechanical Engineering, Faculty of Science and Technology, Keio University, 3-14-1 Hiyoshi, Kohoku-ku, Yokohama 223-8522, Japan.

E-mail: oakiyuya@applc.keio.ac.jp

Contents

Experimental methods S-2
Combinatorial polymerization experiment (Figure S1) S-5
FT-IR spectra of the reference compounds (Figure S2) S-7
^{13}C NMR spectra (Figure S3) S-8
UV-Vis-NIR spectra (Figure S4) S-9
Estimated composition (Table S1) S-10
Composition of the amorphous CPNs in the previous works (Scheme S1) S-11
XRD patterns of the reference materials (Figure S5) S-12
Pore-size analyses (Figures S6 and S7) S-13
MD simulation of NQ-DVB (Figure S8) S-14
MD simulation of reference graphene (Figure S9) S-15
Thickness estimated from the MD simulation (Figure S10) S-16
Exfoliation of NQ-DVB (Figure S11) S-17
High-resolution TEM images of the exfoliated nanosheets (Figure S12) S-18
Structural analyses of the thinner exfoliated nanoflakes (Figure S13) S-19
NQ-DVB extracted from the composite (Figure S14) S-20
Mechanical properties and morphologies of PS/NQ-DVB (Figure S15) S-21
Mechanical properties of the other reference composites (Figure S16) S-22
Mechanical properties in previous works (Table S2 and Figure S17) S-23

Experimental methods

Synthesis of NQ-DVB and TC-DVB. The mixture of NQ powder (TCI, 98.0 %, 323 mg (2.00 mmol)) and DVB liquid (TCI, *meta* and *para* mixture, 0.572 cm^3 (2.00 mmol)) was put in a glass vial (10 cm^3) with toluene (Kanto, 99.5 %, 1.5 cm^3). The mixture of TC powder (TCI, 98.0 %, 204 mg (1.00 mmol)) and DVB liquid 0.286 cm^3 (1.00 mmol)) was put in a glass vial (10 cm^3) with toluene (1.5 cm^3). These mixtures were heated 200 °C for 1 h under the microwave irradiation (Anton Paar, Monowave 400). The resultant solid was vacuum-dried at 200 °C for 16 h to remove the remaining monomers and oligomers. The method for screening experiment selecting the monomers was described in Figure S1.

Exfoliation of NQ-DVB and TC-DVB. The resultant NQ-DVB and TC-DVB (10 mg) were dispersed in chlorobenzene (20 cm^3, TCI 98.0 %) at 60 °C for 5 min for checking the exfoliation behavior (Figure 4b,c) and 1 h (Figure 4d–f) using an ultrasonic homogenizer (Branson, 250 W). Afte the dispersion, the bulk precipitate was removed using a syringe filter (Whatman, 5 μm in the pore size). When the thinner nanosheets were collected, the dispersion liquid was centrifuged at 6000 rpm for 30 min to remove the larger precipitates. The transparent colloidal liquids were dropped on a cleaned silicon substrate for SEM and AFM observations and collodion membrane for TEM observation.

Syntheses of NQ-DVB with PS. The polymer solution containing 3.15 g PS (Aldrich, Mw = 1.92 $\times 10^5$) was prepared with 27 cm^3 toluene in a glass vessel (50 cm^3, 34 mm in diameter and 65 mm in height). Then, 74.8 mg NQ (0.463 mmol) and 0.0662 cm^3 DVB (0.462 mmol) were added to the polymer solution (2.0 cm^3) to adjust the NQ-DVB concentration 30 wt%. The precursor solution was heated without sealing in a fume chamber using temperature-controlled stage at 200 °C for 5 h. The bulk solid was obtained with evaporation of toluene. The sample was vacuum-dried at 60 °C for 48 h.

Reference samples. The following carbon samples were used as the reference: graphite (Kanto), GC (glassy carbon, Aldrich 99.95 %), r-GO (graphene nanoplatelets, 2–10 nm thickness and 5 μm in width, Fuji-Wako), GO (NiSiNa materials), carbon nanotube (multiwalled, 6–13 nm width and 2.5–20 μm in length, Aldrich 98 %), and PPy (Aldrich).

Structural characterization. An increase in the molecular weight with the polymerization was anaylzed using TG under air atmosphere (Shimadzu DTA-60) with 10 K min^{-1}. The molecular structures were studied using FT-IR (Jasco FT-IR 4200) with KBr method and ^{13}C NMR (JEOL JNM-ECZ400R) with CP/MAS method. UV-Vis-NIR spectra were measured by

diffuse-reflectance method using a spectrophotometer with an integrated sphere (Jasco V-670). Raman spectra were obtained with excitation at 533 nm (Renishaw In Via Raman). The crystallinity was measured by XRD using a silicon sample holder without the reflection (Bruker D8 Advance). The morphology observation was carried out using SEM (JEOL JSM-7100-F and Carl Zeiss MERLIN VP compact) operated at 5.0 kV, TEM (FEI Tecnai G2) operated at 200 kV, and AFM (Shimadzu SPM-9700HT). The particle-size distribution was measured using DLS (Ohtsuka Electronics ELSZ-2000). The nanoscale porous structure was analyzed using PALS (Toyo Seiko Type LII) and nitrogen adsorption-desorption method (Shimadzu 3Flex). The composition was measured using CHN elemental analysis (Elementar Japan Unicube).

Mechanical properties. The PS/NQ-DVB composite was compressed using a tester (Shimadzu EZ-LX). The compression stress 1000 N was applied to the bulk composite (16 mm in diameter and 5 mm in height) using a metallic probe with the contact area 2 mm in diameter by the compression rate 10 cm min^{-1}.

MD simulation. All the expanded networks and their stacked structures were simulated by GROMACS[S1] using the second-generation general AMBER force field (GAFF2).[S2] The TC-DVB and NQ-DVB unit layers were prepared using 5571 and 3958 atoms with preserving the compositions in Scheme 1, respectively (Figure 3c and Figure S8). For reference, ideal graphene was prepared with 1015 atoms (Figure S9). One unit layer, 0.5 nm for NQ-DVB and TC-DVB and 0.3 nm for graphene, was stacked to construct the initial structure consisting of 10 layers with a 90 ° rotation to eliminate initial coordinate dependence. A constant-volume and temperature simulation was performed after energy minimization using the steepest descent method for this 10-layer structure. The Bussi-Donadio-Parrinello thermostat was used to control the temperature during MD simulations.[S3] Then, cyclohexane molecules were filled into the edge space of the cell to avoid unrealistic deformation of the layers. Next, constant-pressure and -temperature simulation was performed for this structure at 0.1 MPa and 60 °C. The compressibility in the *x*- and *y*- directions was set to 0 so that the box size did not change in the *x*- and *y*- directions.

Additional Reference

S1. Berendsen, H. J. C.; Spoel, D. V. D.; Drunen, V. R. GROMACS: A message-passing parallel molecular dynamics implementation. *Comp. Phys. Commun.* **1994**, *91*, 43–56.

S2. Wang, J.; Wolf, M. R.; Caldwell, W. J.; Kollman, A. P.; Case, A. D. Development and testing of a general amber force field. *J. Comp. Chem.*, **2004**, *25*, 1157–1174.

S3. Bussi, G.; Donadio, D.; Parrinello, M. Canonical sampling through velocity rescaling. *J. Chem. Phys.* 2007, **126**, 014101.

Combinatorial polymerization experiment

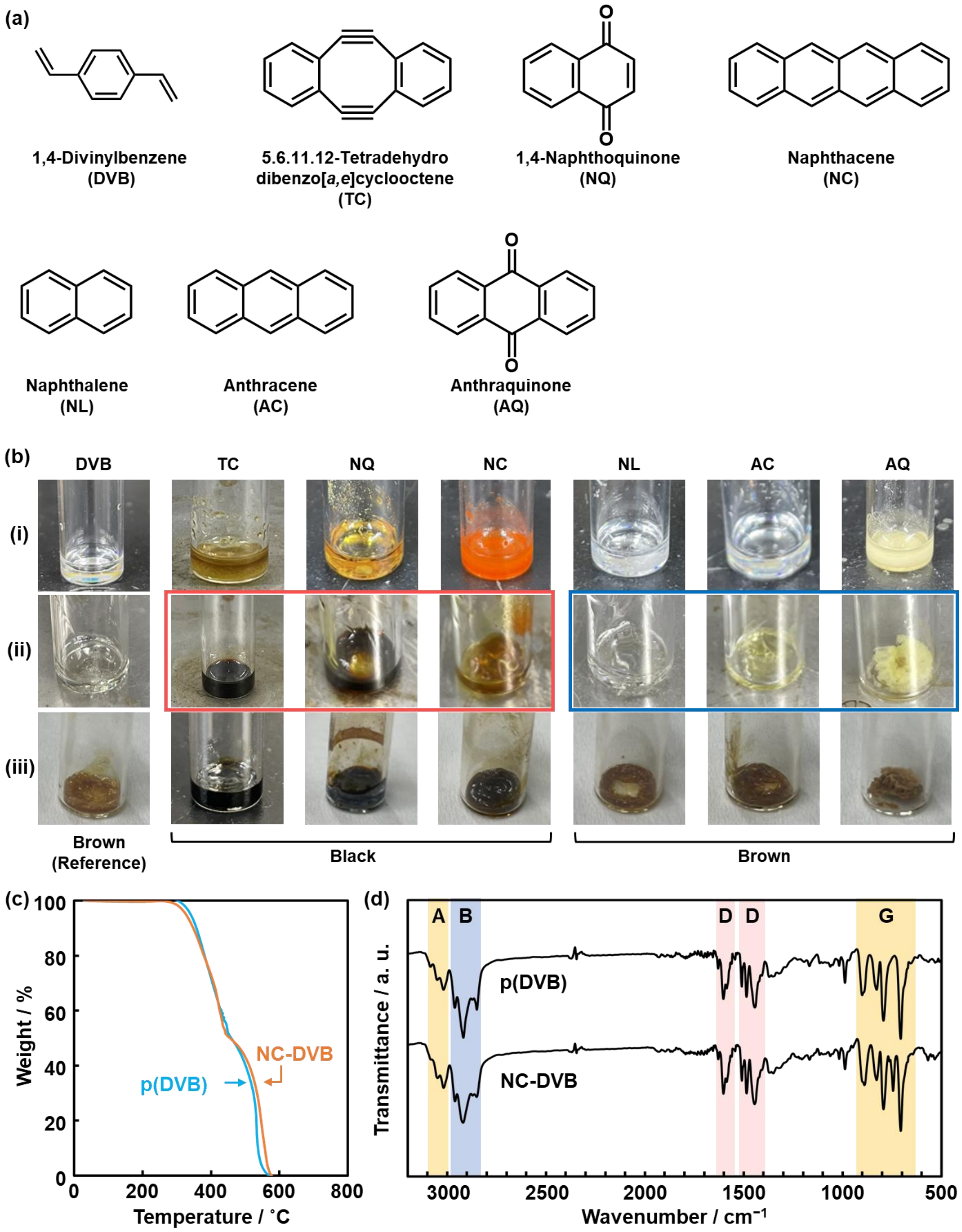

Figure S1. Combinatorial polymerization experiment for screening the combinations of the monomers. (a) Molecular structures of monomers. (b) Photographs of the precursor solution (i), its after heating (ii), and its subsequent vacuum drying (iii). (c,d) TG curves (c) and FT-IR spectra (d) of p(DVB) and NC-DVB.

DVB (0.1 mmol) and the following dienophiles (0.1 mmol) were mixed (Figure S1a): TC, NQ, naphtacene (NC, TCI 97.0 %), naphthalene (NL, Kanto 99.0 %), anthracene (AT, TCI 97.0 %), and anthraquinone (AQ, Kanto 99.0 %). The equimolar mixtures without solvent in a sealed glass vessel (2 cm^3) were heated stepwise in the range of 60–200 °C: 60 °C for 3 min, 80 °C for 3 min, 100 °C for 3 min, …, and 200 °C for 3 min under ambient pressur using a temperature-controlled stage. After heating at 200 °C, all the sample bottles were dried under vacuum condition at 200 °C for 16 h to remove the monomers and oligomers.

If the samples show the color changes after the heating ((ii) in Figure S1b), the polymerized compounds can be obtained by the reaction. The dark colors, such as black and brown, imply the polymerization with extension of the conjugation length. Whereas TC, NQ, and NC showed the color change to black after the reaction with DVB (red frames in Figure S1b), no color change was observed for NP, AT, and AQ (blue frames in Figure S1b). The screening implies the polymerization of TC, NQ, and NC with DVB.

The polymerization of DVB itself was carried out as a reference. NC-DVB and the polymerized DVB (p(DVB)) showed the same FT-IR spectra including the following absorptions (Figure S1d): C–H stretching vibrations of aromatic ring around 3050 cm^{-1} (band A) and methylene around 2950 cm^{-1} (band B), C=C stretching vibrations of the aromatic ring in the range of 1400–1600 cm^{-1} (band D), C–H in-plane bending vibration of the aromatic ring (band G). The TG curves of NC-DVB and p(DVB) showed the same behavior of the weight loss in the lower temperature region compared with that of TC-DVB and NQ-DVB (Figure S1c). These facts indicate that NC and DVB were not copolymerized by the microwave synthesis (Figure S1c,d).

TG curves and FT-IR spectra of the reference compounds

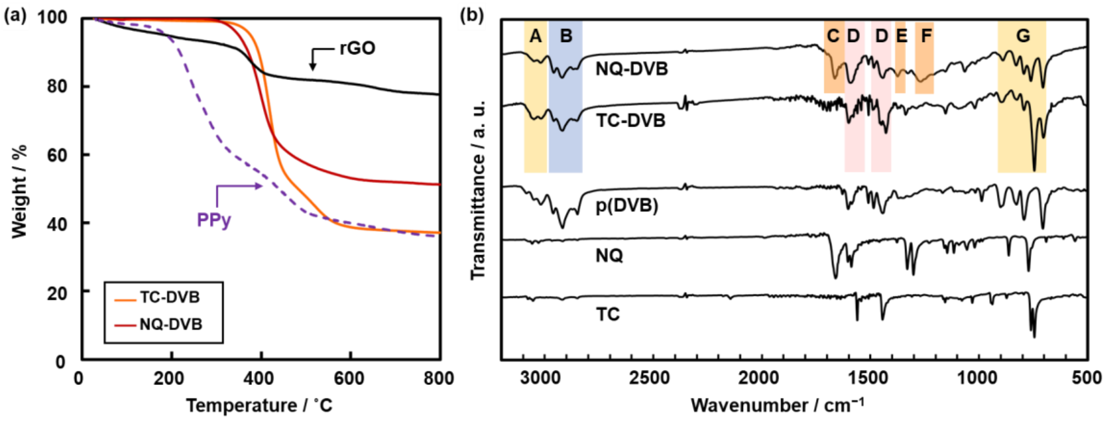

Figure S2. TG curve under Ar atmosphere (a) and FT-IR spectra of TC-DVB, NQ-DVB, and their the refence pDVB, TC monomer, and NQ monomer (b).

TG analysis was carried out under Ar atmosphere with heating at 10 K min^{-1} (Figure S2a). The thermal decomposition of TC-DVB and NQ-DVB around 400 °C was similar to that of rGO. In contrast, the chain-like PPy showed the thermal decomposition at the lower temperature. TC-DVB and NQ-DVB showed the FT-IR spectra different from those of the reference p(DVB) and monomers (TC and NQ) (Figure S2b).

^{13}C NMR spectra

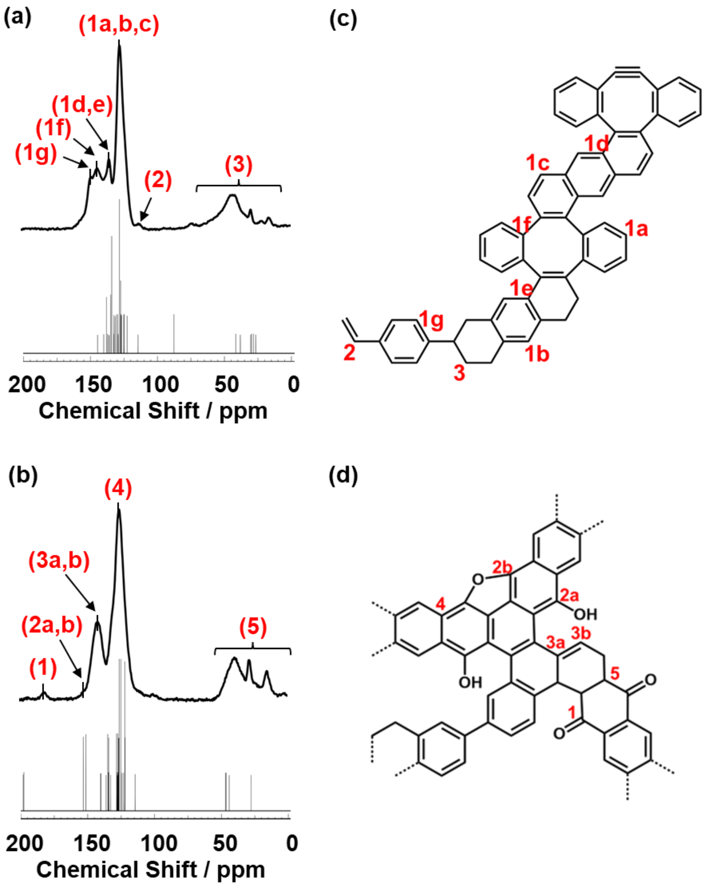

Figure S3. ^{13}C NMR spectra (a,b) and partial oligomeric structures (c,d) of TC-DVB (a,c) and NQ-DVB (b,d).

The solid powder of TC-DVB and NQ-DVB was used for the ^{13}C magic-angle spinning (MAS)-NMR spectra. The broadened spectra characteristic of polymer were obtained for both TC-DVB and NQ-DVB (Figure S3a,b). The spectra were simulated from the partial oligomeric structures (Figure S3c,d). If the simulated peaks are broadened and integrated, the spectra can be consistent with the observed spectra (the bottom bars in Figure S3a,b). Therefore, the NMR analysis indicate the formation of the estimated structures.

UV-Vis-NIR spectra

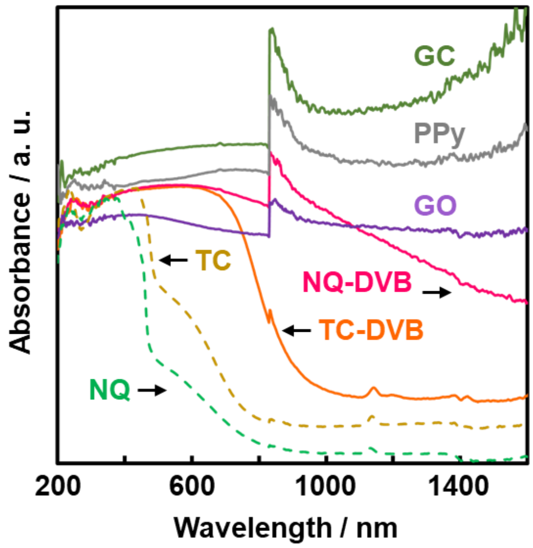

Figure S4. UV-Vis-NIR spectra of TC-DVB, NQ-DVB, and their related samples.

The spectra were measured by diffuse reflectance of the solid powders using an integrated sphere. The noise at 800 nm was caused by changes in the right source. TC-DVB and NQ-DVB showed the expansion of the absorption edge to the NIR region whereas the TC and NQ monomers had no absorption in such range. The absorption in the entire range of UV-Vis-NIR was observed for the reference samples, such as GC, PPy, and GO, with the fully extended π-conjugated system. These results indicate that the conjugation length of TC-DVB and NQ-DVB is extended by the polymerization.

Estimated composition

Table S1. CHN elemental analysis of TC-DVB and NQ-DVB.

TC-DVB	C / wt %	H / wt %	N / wt %	Other (O) / wt %
Sample (i)	92.47 ± 0.19	6.01 ± 0.07	0.12 ± 0.01	1.40 ± 0.27
Sample (ii)	92.84 ± 0.23	6.15 ± 0.09	0.12 ± 0.00	0.89 ± 0.19
Sample (iii)	92.76 ± 0.18	6.12 ± 0.07	0.12 ± 0.01	1.00 ± 0.33
Average	92.69 ± 0.35	6.09 ± 0.17	0.12 ± 0.01	1.10 ± 0.47
Calculated	92.73	6.13	0.00	1.14
NQ-DVB	C / wt %	H / wt %	N / wt %	Other (O) / wt %
Sample (i)	86.73 ± 0.19	6.19 ± 0.02	0.42 ± 0.02	6.66 ± 0.20
Sample (ii)	86.40 ± 0.15	6.16 ± 0.03	0.41 ± 0.01	7.03 ± 0.25
Sample (iii)	86.40 ± 0.18	6.18 ± 0.02	0.45 ± 0.02	6.97 ± 0.19
Average	86.51 ± 0.30	6.18 ± 0.04	0.43 ± 0.03	6.89 ± 0.39
Calculated	86.73	5.89	0.00	7.38

In CHN elemental analysis, the three samples synthesized in the different batches were analyzed to ensure the reproducibility related to the synthetic processes (samples (i)–(iii) in Table S1). In addition, the measurement was carried out three times for each sample to ensure the reproducibility in the analysis.

The estimated structural units in Scheme 1 were drawn based on the results of FT-IR and NMR (Scheme 1). The percentage of water was estimated from the weight loss lower than 200 °C in the TG curves (Figure 2a). The compositions were calculated from the average weight ratio of the CHN elemental analysis in Table S1 (measured value). Then, the weight ratio of the C, H, and N was calculated from the estimated structures in Scheme 1 for the validation (calculated value). The average measured and calculated values are consistent with each other within 0.5 wt %.

Composition of the amorphous CPNs in the previous works

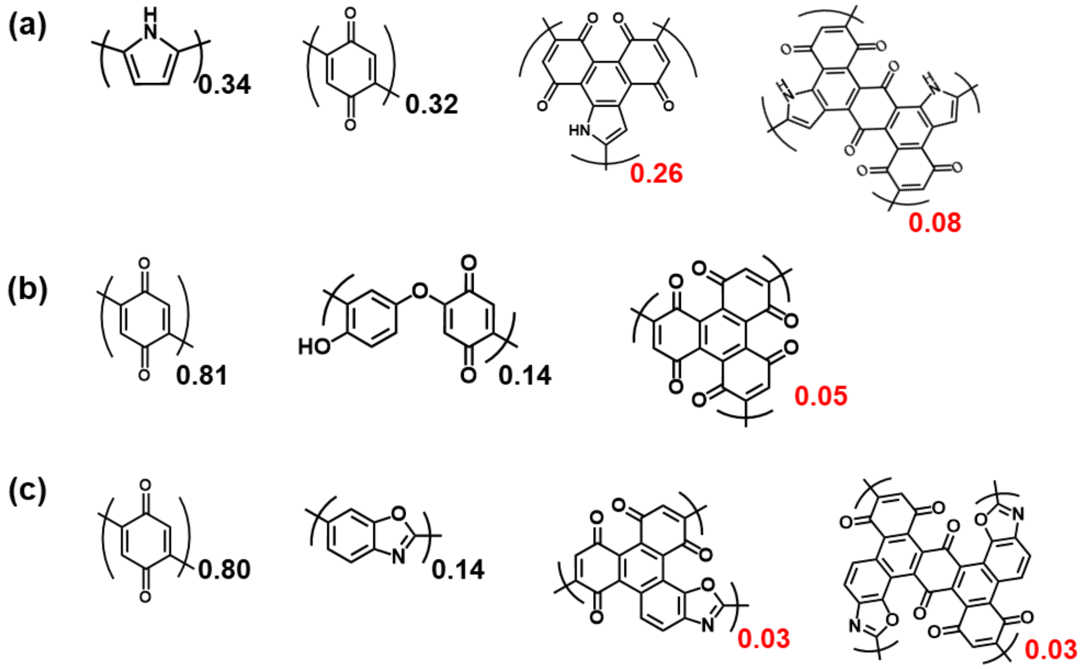

Scheme S1. Estimated structural units and their compositions of BQ-Py (a), BQ-HQ (b), and BQ-BO (c).[33–35]

In the network structures, TC-DVB and NQ-DVB contained 70 % and 59 % of the expanded conjugated domains, respectively (the red colored content in Scheme 1). On the other hand, the ratio of the conjugated domains was low for the other amorphous CPNs: 34 % for BQ-pyrrole (Py),[33] 5 % for BQ-hydroquinone (HQ),[34] and 6 % for BQ-benzoxazole (BO) (Scheme S1).[35] These results indicate that the graphitic domains are expanded in the network structures of TC-DVB and NQ-DVB.

XRD patterns of the reference materials

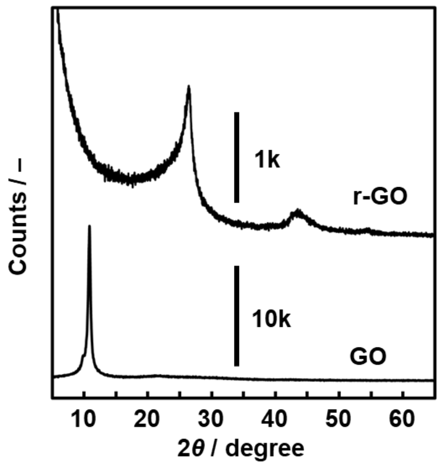

Figure S5. XRD patterns of a commercial r-GO and GO.

TC-DVB and NQ-DVB showed the more broadened peaks compared with those of these reference carbon samples (Figure 2e).

Pore-size analyses

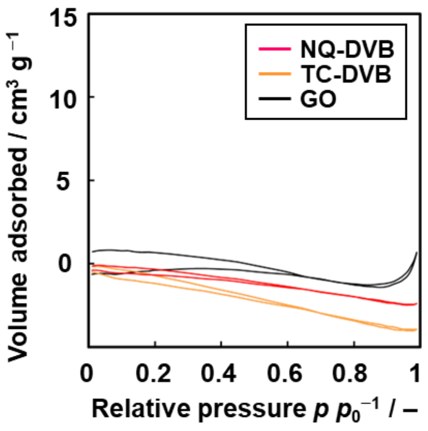

Figure S6. Nitrogen adsorption-desorption isotherms of TC-DVB, NQ-DVB, and GO.

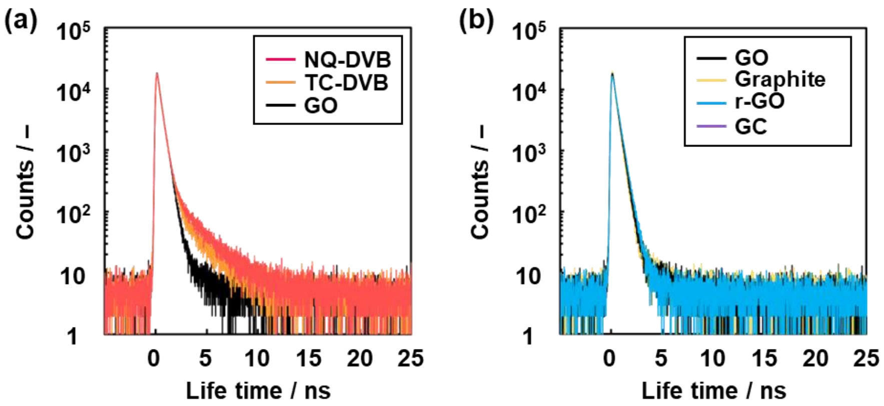

Figure S7. PALS life-time curves of TC-DVB, NQ-DVB, and their reference samples.

Nitrogen adsorption-desorption isotherms indicate that NQ-DVB, TC-DVB, and GO had no porous structure related to the gas adsorption (Figure S6). PALS life-time curves were collected to achieve total 10^6 counts (Figure S7). The life-time curves were analyzed by three components to compare the nanostructures (Table 1).

MD simulation of NQ-DVB

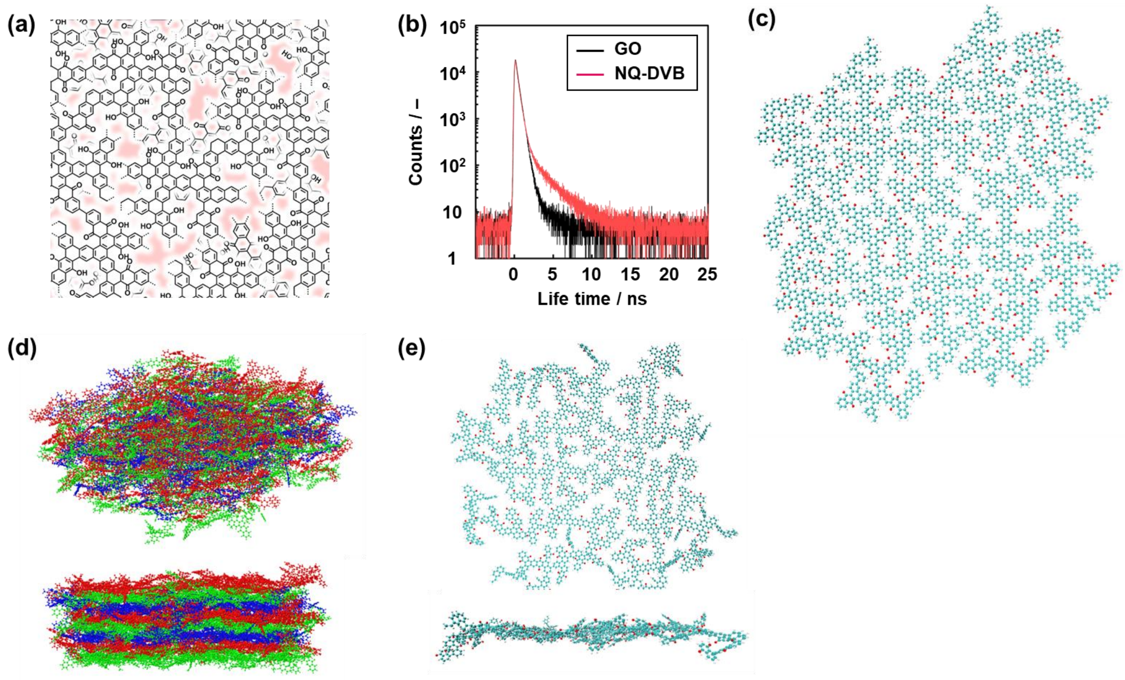

Figure S8. Structural analyses and MD simulation of the NQ-DVB network and its stacking. (a) Schematic illustration. (b) PALS life-time curves of NQ-DVB and reference GO. (c) Original unit layer for the calculation. (d) Diagonal (upper) and side (lower) views of the stacked structure including second-to-eighth layers after the calculation. (e) Top (upper) and side (lower) views of the monolayer after the calculation.

The MD simulation of NQ-DVB showed the similar results as those for TC-DVB in the main text. The uneven monolayers of NQ-DVB network were interpenetrated and stacked with each other. (Figure S8).

MD simulation of reference graphene

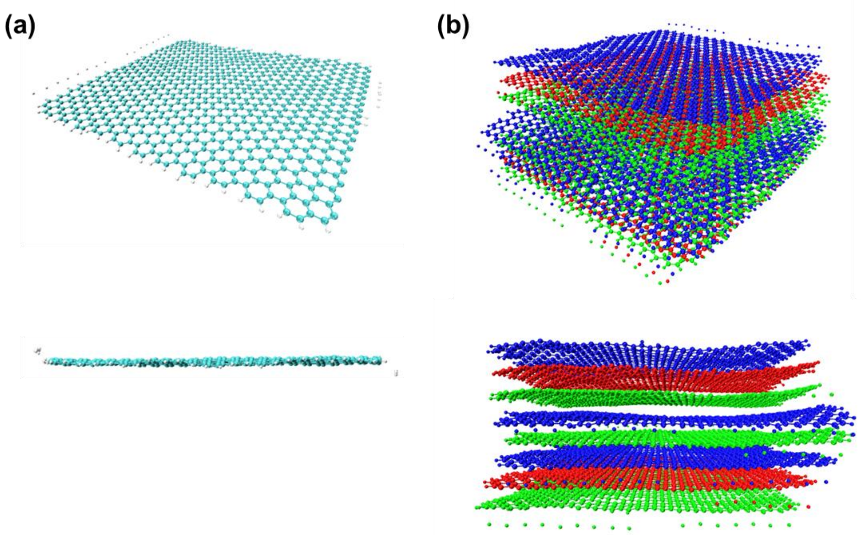

Figure S9. MD simulation of the reference graphene. (a) Top (upper) and side (lower) views of the monolayer after the calculation. (e) Diagonal (upper) and side (lower) views of the stacked structure including second-to-eighth layers after the calculation.

The reference ideal graphene had the flat structure and its stacking (Figure S9). In addition, the calculated interlayer distance 0.333 nm is consistent with that of graphite and turbostratically stacked graphene layers. The reference data supports the validity of this simulation.

Thickness estimated from the MD simulation

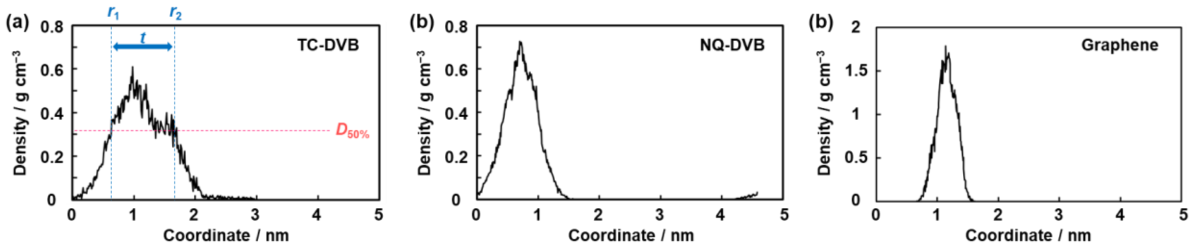

Figure S10. Representative relationship between the coordinate and density of TC-DVB (second layer) (a), NQ-DVB (second layer) (b), and reference graphene (fourth layer) calculated from the results of MD simulation.

After the calculation, one layer was visualized from the stacked states to estimate the thickness. Viewed from the side, the relationship between the coordinate and density was calculated on each layer (Figure S10). As shown in Figure S10a, the calculated thickness (t / nm) was defined as the difference in the coordinates ($t = r_2 - r_1$) within the range of density higher than 50 % of the maximum value ($D_{50\%}$). For example, t was calculated to be 1.06 nm for TC-DVB (Figure S10a), 0.584 nm for TC-DVB (Figure S10b), and 0.380 nm for reference graphene (Figure S10c). The thickness was calculated for the second to eight layers. The average thickness of the monolayers was calculated to be 0.94 ± 0.19 nm for TC-DVB, 0.53 ± 0.04 nm for NQ-DVB, and 0.39 ± 0.02 nm for reference graphene. After the exfoliation and purification, the thin nanosheets less than 1 nm were observed for TC-DVB and NQ-DVB (Figure S12).

Exfoliation of NQ-DVB

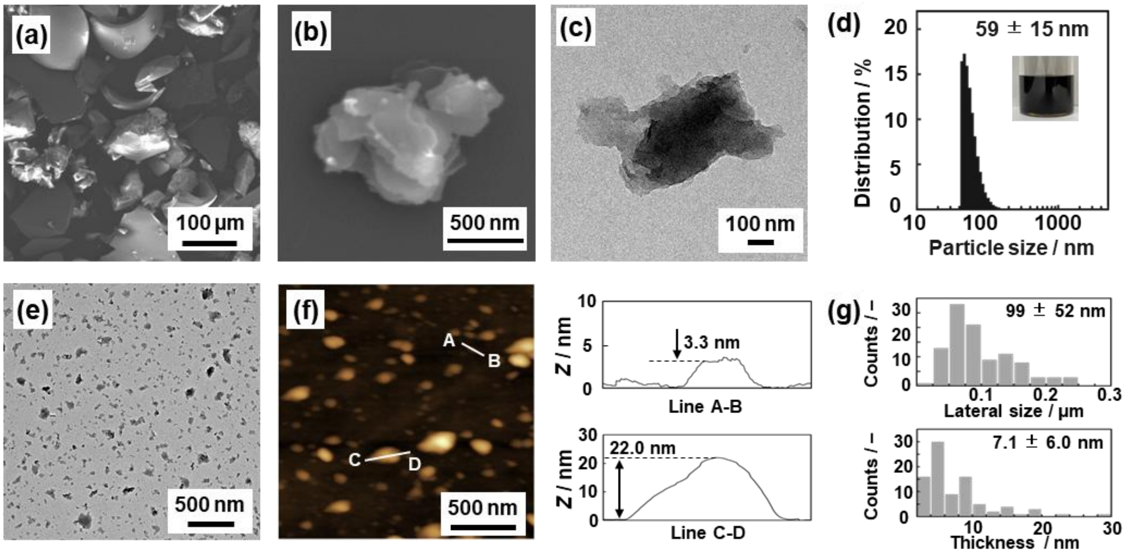

Figure S11. Morphology of NQ-DVB before and after the exfoliation for 5 min (panel b,c) and 60 min (panels d–f). (a) SEM image after the synthesis. (b, c) SEM and TEM images after the exfoliation in chlorobenzene under sonication for 5 min, respectively. (d) DLS particle-size distribution and photograph of the dispersion liquid (inset) after the exfoliation for 1 h. (e) TEM image. (f) AFM image (left) and representative height profiles (right) of the thinner (upper) and thicker (lower) nanoflakes. (g) Distribution of the lateral size (upper) and thickness (lower) estimated from TEM and AFM images, respectively.

As observed in TC-DVB (Figure 4), the similar NQ-DVB nanosheets were obtained by the exfoliation in chlorobenzene within 1 h.

High-resolution TEM images of the exfoliated nanosheets

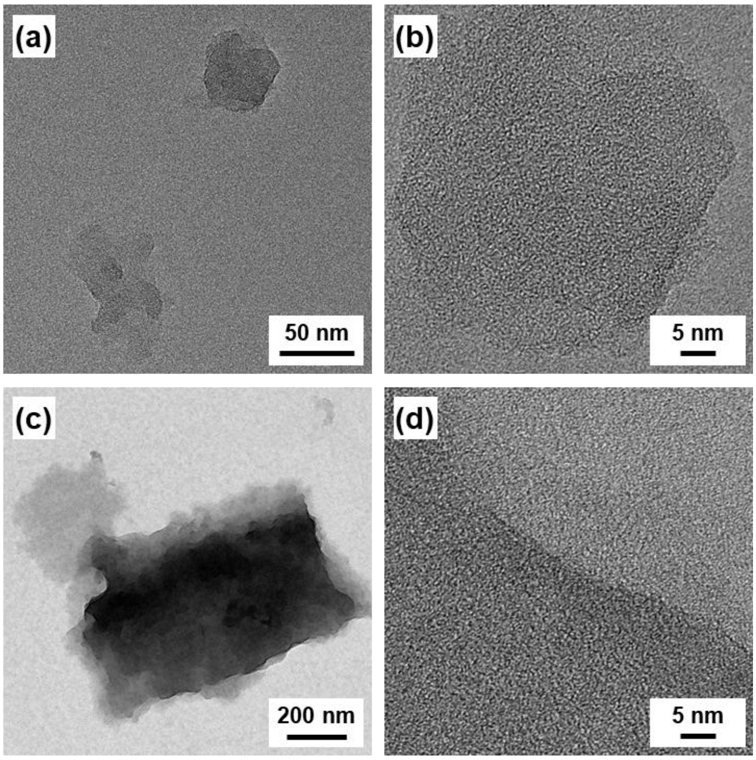

Figure S12. TEM (a,c) and magnified high-resolution TEM (b,d) images of TC-DVB (a,b) and NQ-DVB (c,d) exfoliated nanosheets.

The lattice fringes corresponding to the crystalline graphitic structures were not observed on these images (Figure S12). When the observation was carried out at the higher magnification, the lattice fringes corresponding to graphitic structure appeared by the damages of the electron-beam irradiation. These observations indicate that the original TC-DVB and NQ-DVB nanosheets had the amorphous interior structures as shown in Figure 3a.

Structural analyses of the thinner exfoliated nanoflakes

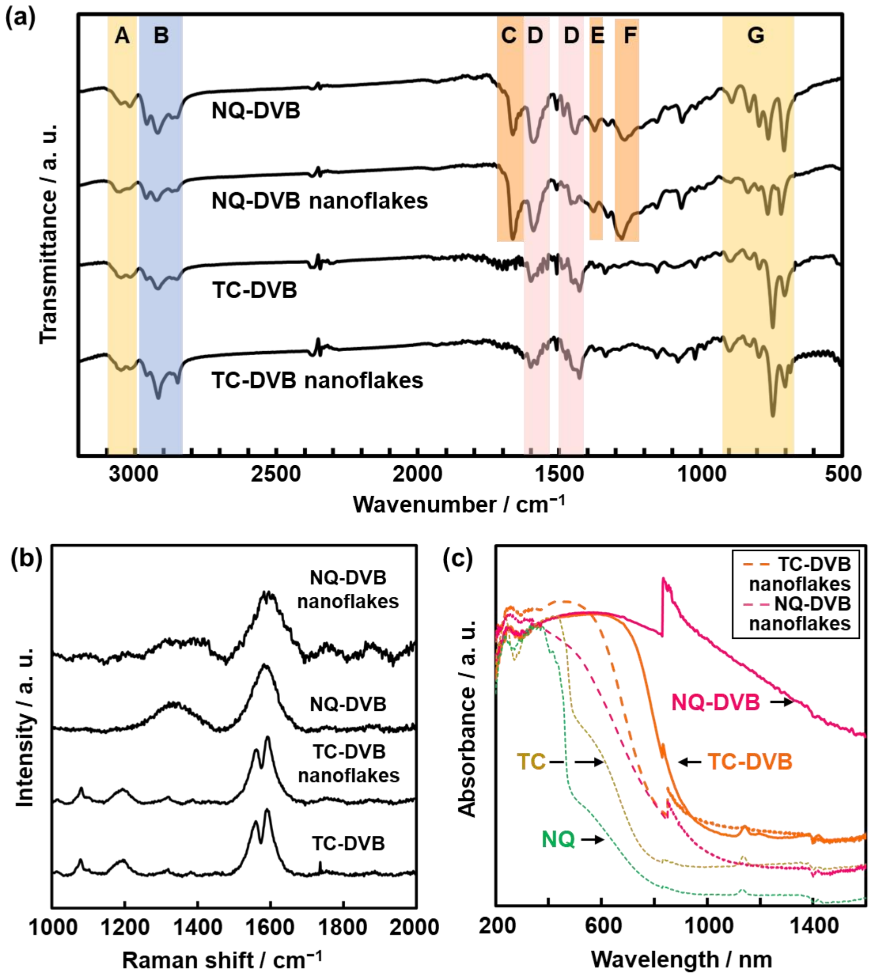

Figure S13. Structural analyses of the stacked TC-DVB and NQ-DVB and their nanoflakes after the exfoliation and subsequent removal of the bulky precipitates. (a) FT-IR spectra. (b) Raman spectra. (c) UV-Vis-NIR spectra.

The powdered samples were obtained by evaporation of the solvent from the dispersion liquids containing the nanoflakes after exfoliation and centrifugation. The FT-IR and Raman spectra showed no changes in the peak positions for the precursor materials and their exfoliated nanoflakes (Figure S13a,b). On the other hand, the absorption edges were shifted to the shorter wavelength region for the exfoliated thinner nanoflakes (Figure S13c). The results indicate that the molecular structure is not changed for the nanoflakes after the exfoliation. As the thinner nanoflakes based on the amorphous network structures afford the motion in the dispersion media, the torsion of the conjugated networks induces the shortening the effective conjugation length.

NQ-DVB extracted from the composite

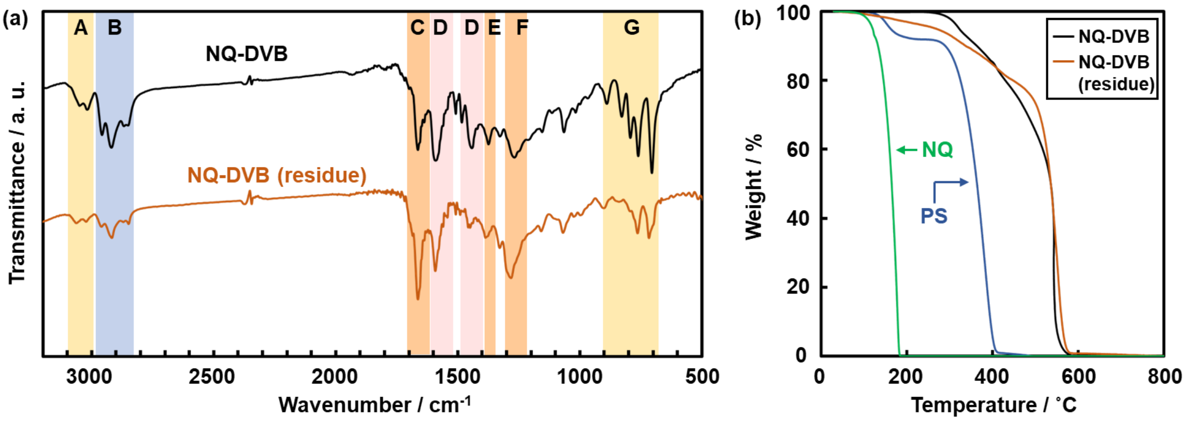

Figure S14. Structural analyses of NQ-DVB extracted from the PS/NQ-DVB composite (residue). (a) FT-IR spectra. (b) TG curves.

The residual NQ-DVB was extracted from the PS/NQ-DVB composite with the immersion in toluene to dissolve PS at 60 °C for 1 h. The residual NQ-DVB showed the FT-IR spectrum and TG curve similar to that of the original one. The results indicate the formation of NQ-DVB in the presence of PS in the precursor solution.

Mechanical properties and morphologies of PS/NQ-DVB

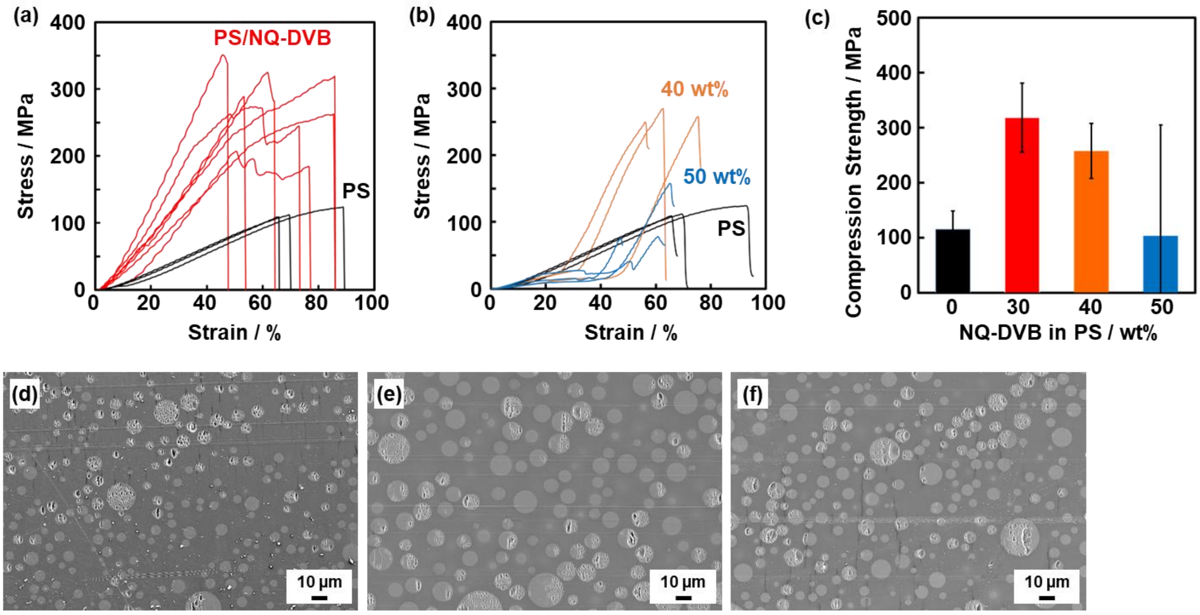

Figure S15. Mechanical properties and morphologies of PS/NQ-DVB (30 %). (a) Stress-strain curves of PS ($N = 3$) and PS/NQ-DVB ($N = 8$). (b) Stress-strain curves of PS and PS/NQ-DVB at the NQ-DVB concentration 40 ($N = 3$) and 50 % ($N = 3$). (c) Relationship between the NQ-DVB concentration and maximum compression strength. (d–f) Cross-sectional SEM images of PS/NQ-DVB at the NQ-DVB concentration 30 (d), 40 (e), and 50 (f) %.

The mechanical properties of PS/NQ-DVB had the reproducibility and significant differences with those of PS (Figure S15a). The mechanical properties were not improved with increasing the NQ-DVB concentration to 40 % and 50 wt% (Figure S15b,c). The size of the island domains corresponding to NQ-DVB increased with increasing the NQ-DVB concentration (Figure S15d–f).

Mechanical properties of the other reference composites

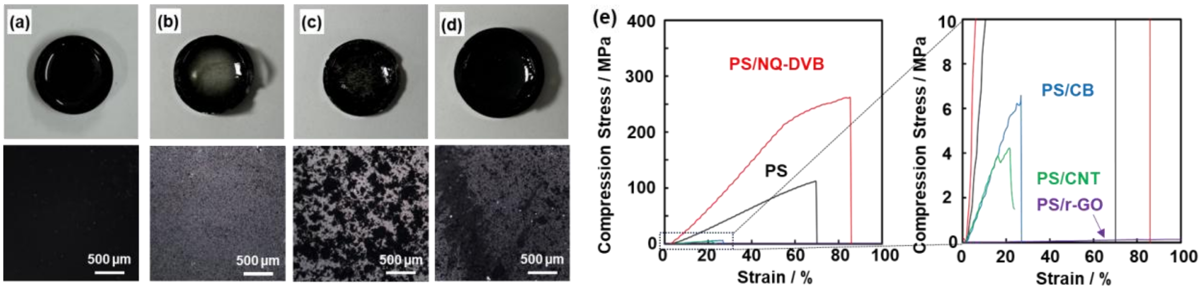

Figure S16. Photographs (a-d) and stress-strain curves (e) of the reference composites. (a–d) Photographs (upper) and optical microscopy images (lower) of the PS containing 30 wt-% NQ-DVB (a), 1 wt-% CB (b), 1 wt-% CNT (c), 1 wt-% r-GO (d). (e) Stress-strain curves and their magnified ones (right panel) of the reference PS/CB, PS/CNT, and PS/r-GO composites.

The homogeneous composites were not obtained by the other reference nanocarbons (Figure S16a–d). The fillers are not homogeneously dispersed in the PS matrix. The mechanical properties were not improved with addition of these reference nanocarbons (Figure S16e).

Mechanical properties in previous works

Table S2. Mechanical properties of reinforced plastics using fillers.

No	Composites	Filler / wt%	Compression strength σ / MPa	Increasing rate / σ / σ_0	Ref
–	PS/NQ-DVB	30	318	2.76	Present work
		40	276	2.39	
		50	104	0.90	
1	Carbon nanofiber (CNF)/glass fiber (GF)/polyester	0.1	107.0	1.46	67
		0.2	115.7	1.60	
		0.3	95.4	1.36	
2	MWCNT/epoxy (with dispersant)	0.5	795	1.41	68
		1	715	1.27	
	MWCNT/epoxy (without dispersant)	0.5	670	1.19	
		1	640	1.13	
3	Graphene/carbon fiber (CF)/epoxy	0.1	298.0	1.27	69
		0.3	358.6	1.53	
		0.5	321.1	1.37	
	Graphene (NH_2-modified) /CF/Epoxy	0.1	380.6	1.62	
		0.3	353.3	1.51	
		0.5	355.4	1.52	
	Graphene (COOH-modified)/CF/Epoxy	0.1	271.5	1.16	
		0.3	414.5	1.77	
		0.5	350.2	1.49	
	Graphene (OH-modified)/CF/Epoxy	0.1	331.1	1.41	
		0.3	311.8	1.33	
		0.5	335.4	1.43	
4	GO/CF/epoxy	0.025	157.0	1.11	70
		0.050	163.5	1.15	
		0.075	171.5	1.21	
		0.100	159.5	1.12	
	GO(-COOH modified)/CF/epoxy	0.025	157.5	1.11	
		0.050	163.0	1.15	
		0.075	170.0	1.20	
		0.100	161.5	1.14	
5	Graphite/CF/Epoxy	3	608	1.10	71
		5	638	1.16	
6	Graphene/hydroxyapatite /polyamide(PA) 66	1	93.7	1.29	72
7	Silica/epoxy	2	91.6	1.03	73
		4	91.5	1.03	
		6	91.9	1.03	
		8	92.3	1.04	
		10	91.1	1.02	
		20	94.8	1.06	
	Halloysite/Epoxy	2	90.8	1.02	
		5	89.3	1.00	
		7	90.9	1.02	
		10	90.4	1.01	
		15	91.3	1.02	
	Liquid rubber (CTBN)/epoxy	5	92.4	1.04	
		10	89.3	1.00	

	Halloysite/CTBN/epoxy	10+5	93	1.04	
	Silica/CTBN/epoxy	10+5	96.2	1.08	
		10+10	90.1	1.01	
8	Silica/CF/epoxy	10	382	1.04	73
		20	388	1.06	
	Halloysite/CFepoxy	5	385	1.05	
		10	449	1.22	
9	GF/polyether-ether-ketone (PEEK)	30	187	1.22	74
		40	238	1.56	
		50	263	1.72	
10	CF/PEEK	25	605	1.57	75
	Sulfonated-CF(SCF)/PEEK	25	460	1.19	
	GO/SCF/PEEK	25	620	1.61	
11	Bagasse fiber/polypropylene (PP)	30	162.0	3.01	76
		40	160.7	2.99	
12	Carbon-powder waste /Epoxy	10	104.5	1.06	77

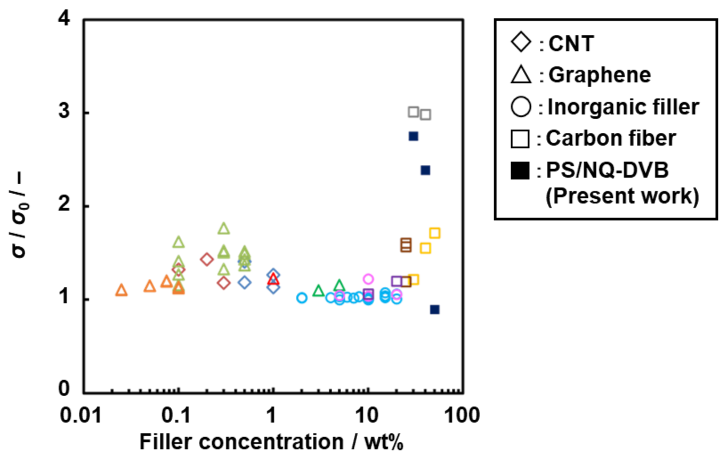

Figure S17. Relationship between the filler concentration and increasing rate of compression strength (σ / σ_0) in previous works.[67–77]

Table S2 and Figure S17 indicate that our PS/NQ-DVB showed both the highest σ and σ / σ_0 compared with those of the other reinforced plastics in previous works.[67–77] Figure S17 was prepared based on Table S2. Although two black squares (□) in Figure S17 showed the higher σ / σ_0 compared with that in the present work (■),[76] σ itself of these samples (162 MPa) was lower than that of our PS/NQ-DVB (318 MPa) in the present work.
